# Debye Temperature Evaluation for Secondary Battery Cathode of α-Sn*_x_*Fe_1−*x*_OOH Nanoparticles Derived from the ^57^Fe- and ^119^Sn-Mössbauer Spectra

**DOI:** 10.3390/ijms25052488

**Published:** 2024-02-20

**Authors:** Ahmed Ibrahim, Kaoru Tani, Kanae Hashi, Bofan Zhang, Zoltán Homonnay, Ernő Kuzmann, Arijeta Bafti, Luka Pavić, Stjepko Krehula, Marijan Marciuš, Shiro Kubuki

**Affiliations:** 1 Department of Chemistry, Graduate School of Science, Tokyo Metropolitan University, Tokyo 192-0397, Japan; elawadyahmed59@gmail.com (A.I.); tani-kaoru1@ed.tmu.ac.jp (K.T.); hashi-kanae@ed.tmu.ac.jp (K.H.); 15054218031@163.com (B.Z.); 2 Institute of Chemistry, Eötvos Loránd University, 1117 Budapest, Hungary; homonnay.zoltan@ttk.elte.hu (Z.H.); erno.kuzmann@ttk.elte.hu (E.K.); 3 Faculty of Chemical Engineering and Technology, University of Zagreb, 10000 Zagreb, Croatia; abafti@fkit.unizg.hr; 4 Division of Materials Chemistry, Ruđer Bošković Institute, 10000 Zagreb, Croatia; lpavic@irb.hr (L.P.); stjepko.krehula@irb.hr (S.K.); marijan.marcius@irb.hr (M.M.)

**Keywords:** tin-goethite nanoparticles, hydrothermal reaction, Mössbauer spectroscopy, secondary battery cathode

## Abstract

Debye temperatures of *α*-Sn*_x_*Fe_1−*x*_OOH nanoparticles (*x* = 0, 0.05, 0.10, 0.15 and 0.20, abbreviated as Sn100*x* NPs) prepared by hydrothermal reaction were estimated with ^57^Fe- and ^119^Sn-Mössbauer spectra measured by varying the temperature from 20 to 300 K. Electrical properties were studied by solid-state impedance spectroscopy (SS-IS). Together, the charge–discharge capacity of Li- and Na-ion batteries containing Sn100*x* NPs as a cathode were evaluated. ^57^Fe-Mössbauer spectra of Sn10, Sn15, and Sn20 measured at 300 K showed only one doublet due to the superparamagnetic doublet, while the doublet decomposed into a sextet due to goethite at the temperature below 50 K for Sn 10, 200 K for Sn15, and 100 K for Sn20. These results suggest that Sn10, Sn15 and Sn20 had smaller particles than Sn0. On the other hand, 20 K ^119^Sn-Mössbauer spectra of Sn15 were composed of a paramagnetic doublet with an isomer shift (*δ*) of 0.24 mm s^−1^ and quadrupole splitting (∆) of 3.52 mm s^−1^. These values were larger than those of Sn10 (*δ*: 0.08 mm s^−1^, ∆: 0.00 mm s^−1^) and Sn20 (*δ*: 0.10 mm s^−1^, ∆: 0.00 mm s^−1^), suggesting that the Sn^IV^-O chemical bond is shorter and the distortion of octahedral SnO_6_ is larger in Sn15 than in Sn10 and Sn20 due to the increase in the covalency and polarization of the Sn^IV^-O chemical bond. Debye temperatures determined from ^57^Fe-Mössbauer spectra measured at the low temperature were 210 K, 228 K, and 250 K for Sn10, Sn15, and Sn20, while that of *α*-Fe_2_O_3_ was 324 K. Similarly, the Debye temperature of 199, 251, and 269 K for Sn10, Sn15, and Sn20 were estimated from the temperature-dependent ^119^Sn-Mössbauer spectra, which were significantly smaller than that of BaSnO_3_ (=658 K) and SnO_2_ (=382 K). These results suggest that Fe and Sn are a weakly bound lattice in goethite NPs with low crystallinity. Modification of NPs and addition of Sn has a positive effect, resulting in an increase in DC conductivity of almost 5 orders of magnitude, from a *σ*_DC_ value of 9.37 × 10^−7^ (Ω cm)^−1^ for pure goethite Sn (Sn0) up to DC plateau for samples containing 0.15 and 0.20 Sn (Sn15 and Sn20) with a DC value of ~4 × 10^−7^ (Ω cm)^−1^ @423 K. This non-linear conductivity pattern and levelling at a higher Sn content suggests that structural modifications have a notable impact on electron transport, which is primarily governed by the thermally activated via three-dimensional hopping of small polarons (SPH). Measurements of SIB performance, including the Sn100*x* cathode under a current density of 50 mA g^−1^, showed initial capacities of 81 and 85 mAh g^−1^ for Sn0 and Sn15, which were larger than the others. The large initial capacities were measured at a current density of 5 mA g^−1^ found at 170 and 182 mAh g^−1^ for Sn15 and Sn20, respectively. It is concluded that tin-goethite NPs are an excellent material for a secondary battery cathode and that Sn15 is the best cathode among the studied Sn100*x* NPs.

## 1. Introduction

Goethite (*α*-FeOOH) is the most stable iron oxyhydroxide available in nature, in which the crystal structure is based on hexagonal closed packing O^2−^ ions with Fe^3+^ located in one-half of the octahedral sites [1,2]. Because of the nontoxicity, stability, and bandgap energy of 2.7 eV in the visible-light range, and its absorption properties, there are various kinds of applications as functional materials for *α*-FeOOH, such as catalysts [3], photocatalysts [4], photoelectrodes [5,6], gas sensors [7,8], absorbents [9,10], and secondary battery electrodes [11,12,13,14,15].

In terms of the increase in the above-mentioned physical properties, nanoparticulation and doping of different elements other than iron are an essential strategy. As for the development of photo-Fenton photocatalytic material, tin-goethite nanoparticles with the formula of *α*-Sn_0.15_Fe_0.85_OOH (Sn15) prepared by hydrothermal reaction exhibited the largest apparent rate constant (*k*) value of 0.035 min^−1^ against methylene blue (MB) in the aqueous solution [16]. By comparing the photocatalytic ability of doping-free goethite (*α*-FeOOH, Sn0) NPs, with a *k* value of 0.024 min^−1^, bandgap energy of 2.47 eV, and specific surface area of 53 m^2^ g^−1^, we could recognize that the high photo-Fenton catalytic ability of Sn15 NPs was provided by the smaller bandgap energy (*E*_g_) of 1.77 eV and the large surface area of 178 m^2^ g^−1^ [16]. This example is one of the successful cases for achieving an increasing surface area and a lowering *E*_g_ by doping different elements into iron oxyhydroxide nanoparticles. Concordant results of photo-Fenton catalytic ability of Ni-substituted goethite (α-Ni*_x_*Fe_1−_*_x_*OOH, Ni100*x*) NPs with an ‘*x*’ of 0.10 showed the surface area of 73.9 m^2^ g^−1^ and the *E*_g_ of 2.55 eV led to the largest *k* value of 0.015 min^−1^ among the studied Ni100*x* [17].

The comprehension of the electronic structure within iron oxides and oxyhydroxides, such as goethite, persists as an ongoing challenge. The goethite structure involves parallel double chains of edge-sharing octahedra. Guskos et al. [18] focused on the electrical properties of goethite and proposed that charge transport occurs through thermally activated three-dimensional hopping of electrons via oxygen vacancies. In such materials, the key to charge transport lies in the Fe^2+^-Fe^3+^ exchange reaction between edge-sharing iron octahedra which facilitates the hopping of small polarons (SPH) across the lattice [19,20]. Depending on the mineral and local charge state, the small polaron can either encase an electron or hole, which in the case of goethite is an electron (e^−^ polaron). Vitaly et al. [21] showed that RT charge transport in goethite is primarily governed by the thermally activated SPH, with various inequivalent paths for electron hopping, mediated by O and OH species, with the mobility being higher compared to other iron oxyhydroxide (FeOOH) polymorphs. In our recent investigation [17], which delved into the electrical properties of Ni*_x_*Fe_1−*x*_OOH NPs across a wide compositional range (*x* = 0–0.5) using impedance spectroscopy, a discernible decrease in DC conductivity was observed with an increase in Ni content. This observation is attributed to the predominant influence of a decrease in Fe content and charge carrier concentration, coupled with an increase in polaron hopping activation energies. Consequently, this leads to a reduction in the ground state carrier mobility compared to pure goethite.

Concerning the development of new cathode active materials for secondary batteries, Ibrahim reported that Ni100*x* NPs with an ‘*x*’ of 0.10 showed the highest first discharge capacity of 363 mAh g^−1^ for the Li-ion battery and that with ‘*x*’ of 0.20 showed that 223 mAh g^−1^ for the Na-ion battery evaluated under 5 mA g^−1^, respectively [17].

In order to achieve the precise structural evaluation of iron- and tin-containing materials, Mössbauer spectroscopy is the most appropriate technique because it applies the largest electromagnetic energy of gamma-ray around 10 ~ 20 keV to irradiate the testing samples for the characterization [22]. Therefore, atomic-level structural change can be detected with Mössbauer spectroscopy. With the help of Mössbauer spectroscopy, the chemical bond between the Mössbauer nucleus, such as iron or tin and surrounding elements, is reflected as a Mössbauer parameter of isomer shift (IS, *δ*) which reflects the 4s electron density respective to the standard material of *α*-Fe at the nuclear site [23]. The local distortion of polyhedral units composed of iron or tin oxides is reflected as quadrupole splitting (QS, ∆) which is associated with the asymmetrical parameter, (*η*_xx_ − *η*_yy_)/*η_zz_*), where *η*_xx_, *η*_yy_, and *η*_zz_ are electric field gradients on the *x-*, *y-*, and *z-*axis, respectively. Further, the width between the 1st and 6th peaks observed from Mössbauer spectra of the iron compounds having magnetic ordering can be estimated as an internal magnetic field (*H*_int_) proportional to the *H*_int_ of *α*-Fe (=33 T) [24]. In addition to the above-mentioned Mössbauer parameters, the Debye temperature (*Θ*_D_), which can be estimated by the temperature dependency of the recoil-less fraction (*f*), is essential information that reflects the rigidness of the Mössbauer nucleus surrounded by different elements [24]. The *Θ*_D_ values are estimated for evaluating the structure of oxides [25], minerals [26], complexes [27], and glasses [28]. However, there are almost no data for the Debye temperatures of nanoparticles obtained using Mössbauer spectroscopy.

As we know, the *Θ*_D_ is a measure of the average vibrational energy of atoms or molecules in a solid material [29,30]. It is correlated to the stiffness of the material’s lattice and influences various physical properties such as thermal conductivity, specific heat capacity, and elastic modulus [30]. In the context of developing new functional nanoparticles with high performance, understanding the relationship between the *Θ*_D_ values of different materials is crucial. By analyzing these values, we can gain insights into the materials’ lattice structures, bonding strengths, and thermal behaviors, which are essential for designing nanoparticles with desired properties [29,30].

For instance, higher *Θ*_D_ indicates stronger interatomic bonds and greater lattice stiffness, which can contribute to the improved mechanical strength and thermal stability of nanoparticles. Based on this knowledge, we can utilize nanoparticles with enhanced durability and resistance to thermal degradation, making them suitable for applications in harsh environments or high-temperature conditions. Additionally, the *Θ*_D_ can impact the phonon spectrum of materials, affecting their electronic and thermal transport properties [31,32]. Tailoring the *Θ*_D_ of nanoparticles can optimize their electrical conductivity, thermal conductivity, and charge carrier mobility, which is crucial for applications such as energy storage devices, catalysis, sensors, and electronic devices [30,31,32].

Having described the background above, in this paper, we will report on the relationship between the local structure of tin goethite nanoparticles with the composition of *α*-Sn*_x_*Fe_1−_*_x_*OOH NPs characterized by ^57^Fe- and ^119^Sn-Mössbauer spectroscopies, mainly focusing on the Debye temperatures. In addition, the electrical transport properties and the cathode active performances of *α*-Sn*_x_*Fe_1−_*_x_*OOH NPs in Li- and Na-ion batteries are investigated.

## 2. Results and Discussion

In the previously published paper, the structure and photo-Fenton catalytic ability of tin-doped goethite with the composition of Sn100*x* NPs was investigated using X-ray diffractometry (XRD), X-ray absorption near-edge spectroscopy (XANES), transmission electron microscopy (TEM), room temperature ^57^Fe-and ^119^Sn-Mössbauer spectroscopies, Brunauer–Emmett–Teller surface area measurement (BET), diffuse reflectance spectroscopy (DRS), and ultraviolet–visible absorption spectroscopy (UV-Vis) [16].

The XRD patterns of Sn100*x* with ‘*x*’ of 0, 0.05, and 0.15 showed peaks only attributed to goethite (ICDD No: 01-084-8278), while the XRD pattern of Sn10 showed almost no peaks due to the amorphous structure of ferrihydrite, and that of Sn20 contained a by-product of FeSnO(OH)_5_ (ICDD No: 00-031-0654) in addition to goethite [16].

^57^Fe-Mössbauer spectra of Sn0 and Sn5 showed a sextet with an isomer shift (*δ*) of 0.37 mm s^−1^, quadrupole splitting (∆) of −0.27 mm s^−1^, and internal magnetic field (*H*_int_) of 33.2 T due to goethite [16]. In contrast, those of Sn10, Sn15, and Sn20 showed a doublet, increasing *δ* from 0.34 to 0.37 mm s^−1^ and ∆ from 0.62 to 0.63 mm s^−1^ due to the superparamagnetic phase [16]. Meanwhile, increasing *δ* from −0.01 to 0.13 mm s^−1^ and decreasing ∆ from 1.15 to 0.53 mm s^−1^ was observed from the ^119^Sn-Mössbauer spectra of Sn100*x* with ‘*x*’ from 0.05 to 0.20 [16]. These results indicate that the Sn^IV^-O chemical bond becomes shorter, while that of Fe^III^-O is longer by introducing tin into goethite, which causes a decrease in the bandgap energy (*E*_g_). The *E*_g_ of 2.77 eV estimated for Sn-free goethite (Sn0) decreased to 1.77 eV recorded for Sn15 in the MB decomposition test.

TEM images of Sn100*x* showed that needle-like particles with an average length of 181 and 61 nm were observed in Sn0 and Sn5, respectively, whereas nanoparticles with the average size of 4.1, 6.4, and 10.2 nm were for Sn10, Sn15, and Sn20, respectively [16]. From the BET measurement, the specific surface area (SSA) of Sn100*x* increased from 53 to 178 m^2^ g^−1^ with Sn content [16]. The photo-Fenton catalytic ability of Sn100*x* using 20 μmol L^−1^ methylene blue aqueous solution and 0.4 mol L^−1^ H_2_O_2_ under visible-light irradiation revealed that Sn15 showed the most prominent apparent-first-order rate constant (*k*) of 35 × 10^−1^ min^−1^ [16].

As for the structural comparison for the Fe ion in *α*-Sn*_x_*Fe_1−_*_x_*OOH NPs, the temperature dependence Mössbauer spectra were measured for *α*-Fe_2_O_3_, *γ*-Fe_2_O_3_, and Fe_3_O_4_ as indicated in Figure S1a–c.

The obtained Mössbauer parameters of *α*-Fe_2_O_3_, *γ*-Fe_2_O_3_, and Fe_3_O_4_ are listed in Tables S1–S3, respectively. As shown in Table S1, the value of *δ* and *H*_int_ increased from 0.368_±0.002_ to 0.503_±0.003_ mm s^−1^, and from 50.50_±0.01_ to 53.45_±0.02_ T with the decrease in the temperature from 300 to 20 K.

In general, the value of isomer shift (*δ*) reflects on the 4*s* electron density at the nuclear site of the Mössbauer element, which can be expressed as [33]:

*δ* = *C* Δ*R*/*R*{|ψ_A_(0)|^2^ − |ψ_s_(0)|^2^} (1)

where *C* is the constant; ∆*R* and *R* are differences; and the average radius of Mössbauer nuclei between excited and ground states |ψ_A_(0)|and |ψ_S_(0)| are the electron density of absorber and standard, respectively. In the case of ^57^Fe-Mössbauer spectroscopy, the value *δ* decreases when the 4*s* electron density at the nuclear site increases because Δ*R* is negative. Meanwhile, in the case of ^119^Sn-Mössbauer spectroscopy, *δ* value increases with 4*s* electron density because Δ*R* is positive. Therefore, the decreasing *δ* values observed from temperature-dependent Mössbauer spectra are primarily due to the effect of second-order Doppler shift. ^57^Fe-Mössbauer spectra of magnetically ordered iron oxides show splitting due to the magnetic interaction shown below [34]:

(2) $$H_{int}=-{g_{I}\;}_{\;}{µ}_{N}B\Delta m_{I}$$

where *g*_I_ is the nuclear factor, which depends on the nuclear spin; *µ*_N_ is the nuclear magneton; *B* is magnetic flux density; and ∆*m*_I_ is a magnetic quantum number (=1 in ^57^Fe). Generally, a *H*_int_ of magnetically ordered iron oxides is observed as the width between the 1st and 6th peak of the sextet in ^57^Fe-Mössbauer spectra and is in proportion to that of α-Fe, i.e., 33 T. Similar to the temperature dependency of *δ*, the increase in *H*_int_ is observed when lowering the temperature of ^57^Fe-Mössbauer spectra because the spin orientation becomes uniform in the ordering due to the exclusion of the thermal vibration. In addition, asymmetry of the surrounding of the Mössbauer nucleus is reflected in the quadrupole splitting (Q.S., Δ), i.e., [34,35]:

(3) $$\Delta =\frac{1}{2}\;e^{2}qQ{\left\{1+{\frac{\eta }{3}}^{2}\right\}}^{\frac{1}{2}}$$

where *e* is the electron charge; *q* is the gradient of the electrostatic field at the nucleus; *Q* is the nuclear quadrupole moment; and *η* is the asymmetry parameter.

*η* is expressed as [35]:

(4) $$\eta =(V_{xx}-V_{yy})/V_{zz}$$

Where *V*_xx_, *V*_yy_, and *V*_zz_ (=*eq*) are the electrical field gradient on the x, y, and z axis, with the order of |*V*_zz_| > |*V*_yy_| > |*V*_zz_|. As for the sextet observed for *α*-Fe_2_O_3_, the ∆ value is determined by subtracting the width between 5th and 6th from that between 1st and 2nd. As a result, the ∆ value of *α*-Fe_2_O_3_ increased from −0.227 to 0.458 mm s^−1^ with a decrease in temperature from 300 to 20 K. In particular, the symbol of ∆ changes from negative to positive between 260 and 240 K because of the Morin transition [36].

Based on the temperature dependency of Mössbauer parameters of *α*-Fe_2_O_3_, we can compare the structural differences of *γ*-Fe_2_O_3_ and Fe_3_O_4_, both of which are known to have inverse spinel structures [37]. As for the temperature-dependent Mössbauer spectra of α-Fe_2_O_3_, for which the parameters are shown in Table S2, an increasing *δ* value from 0.319_±0.004_ to 0.453_±0.004_ mm s^−1^ and *H*_int_ from 50.24_±0.03_ to 52.95_±0.03_ T were observed, which is a similar tendency to that obtained from *α*-Fe_2_O_3_. Different from temperature-dependent ∆ in the Mössbauer spectra of *α*-Fe_2_O_3_, stable ∆ values ranging from 0.019_±0.007_ to 0.000_±0.009_ were observed for *γ*-Fe_2_O_3_.

On the other hand, Mössbauer spectra of Fe_3_O_4_ are composed of two sub-spectra due to tetrahedral (*T*_d_) and octahedral *(O*_h_) sites of iron, shown in Table S3. As for the Fe^III^(*T*_d_) site, increasing the *δ* value from 0.311_±0.01_ to 0.462_±0.01_ mm s^−1^ and *H*_int_ from 49.02_±0.04_ to 51.33_±0.02_ T were observed from the temperature-dependent Mössbauer spectra of Fe_3_O_4_, while *δ* of Fe^II^(*O*_h_) and Fe^III^(*O*_h_) sites increased from 0.655_±0.01_ to 0.970_±0.02_ mm s^−1^ with decrease in temperature, which indicates that the valence state of iron in the tetrahedral sites changed from Fe^III^ to Fe^II^ [37].

Increases in the *H*_int_ values from 49.02_±0.04_ to 51.33_±0.02_ T for a tetrahedral site and from 45.43_±0.06_ to 46.50_±0.10_ T for the octahedral sites were observed from the ^57^Fe-Mössbauer spectra of Fe_3_O_4_ because the spin orientation becomes uniform in the ordering due to the exclusion of the thermal vibration likely due to the *α*- and *γ*-Fe_2_O_3_.

XANES and EXAFS spectra *α*-Fe_2_O_3_, *γ*-Fe_2_O_3_, and Fe_3_O_4_ measured at 250, 150, and 50 K are shown in Figure S2. For the Fe *K*-edge XANES spectra of *α*-Fe_2_O_3_ and *γ*-Fe_2_O_3_, a stable energy of 7109 eV was recorded at the *μ*E value of 0.5 regardless of the temperature, as shown in Figure S2a. These results indicate that the XANES spectra did not detect the change in the oxidation state of the Fe ion. A slightly smaller energy of 7108 eV was observed at the *μ*E value of 0.5 for the XANES spectra of Fe_3_O_4_, regardless of the measured temperature. The smaller energy of 7108 eV observed from the XANES spectra of Fe_3_O_4_ is due to the existence of Fe^2+^, while *α*-Fe_2_O_3_ and *γ*-Fe_2_O_3_ showed a larger energy of 7109 eV because they contain only Fe^3+^ [38,39]. As for the Fe *K*- edge FT-EXAFS spectra of *α*-Fe_2_O_3_, *γ*-Fe_2_O_3_, and Fe_3_O_4_, the first and second with radial distances of 1.4 Å due to Fe-O and 2.6 Å due to Fe-Fe do not change by changing the temperature, as shown in Figure S2b [38,39]. These two peaks are commonly found in *α*-Fe_2_O_3_, *γ*-Fe_2_O_3_, and Fe_3_O_4_. Therefore, it is difficult to determine the oxidation state of the Fe [38,39]. Overall, the relative intensities of each peak increased with the temperature decrease. The increased relative intensities of each peak in EXAFS spectra are due to the increased possibility of the energy transition of the targeting element of Fe by excluding thermal vibration. By comparing the structural information obtained by ^57^Fe-Mössbauer spectroscopy and Fe *K*-edge XAFS spectroscopy, precise structural information is available for ^57^Fe-Mössbauer spectroscopy.

The adsorption–desorption isotherm curve and {*X*(*p*/*p*_0_) − 1}^−1^ vs. *p*/*p*_0_ plot of *α*-Fe_2_O_3_, *γ*-Fe_2_O_3_, and Fe_3_O_4_, are shown in Figure S3. Figure S3a shows the adsorption–desorption isotherm curves of *α*-Fe_2_O_3_, *γ*-Fe_2_O_3_, and Fe_3_O_4_ showing the type II behavior under the IUPAC classification, indicating that each sample contains macropores larger than 50 nm [40,41]. In fact, the estimated average pore sizes of *α*-Fe_2_O_3_, *γ*-Fe_2_O_3_, and Fe_3_O_4_ are 75.3, 56.9, and 63.5 nm, respectively.

At the relative pressure of 1.0, the total volume of adsorbed N_2_ gas, *V*_a_, of 210 cm^3^ g^−1^ was recorded for *γ*-Fe_2_O_3_, which is larger than that of *α*-Fe_2_O_3_ (=78 cm^3^ g^−1^) and Fe_3_O_4_ (=53 cm^3^ g^−1^). The largest *V*_a_ of *γ*-Fe_2_O_3_ is due to the vacant octahedral site. The Brunauer–Emmet–Teller (BET) theory was applied to estimate the iron oxides’ surface area (SSA), which can be expressed as Equation (5) [41], i.e.:

(5) $$\frac{1}{X\;\left[\left(\;\frac{P_{o}}{P}\;\right)-1\right]}=\frac{1}{X_{m}C}+C-\frac{1}{X_{m}\;C}\left(\;\frac{P_{o}}{P}\;\right)\;$$

where *X* is the weight of nitrogen adsorbed at a given relative pressure (*P/P*_0_); *X*_m_ is monolayer capacity, which is the volume of gas adsorbed at standard temperature and pressure (STP) defined as 273 K and 1 atm; and *C* is constant. Once *X*_m_ is obtained, the total surface area *S*_t_ can be determined by the following Equation (6) [42], i.e.:

(6) $$S_{t}=\frac{X_{m}\;N_{s}}{V},\;S_{BET}=\frac{S_{t}}{a}$$

where *S*_t_, *N*, *s*, *V*, *S*_BET_, *a* are the total surface area, Avogadro’s number, a cross-sectional area of the adsorbed gas molecule, the molar volume of adsorbed gas, specific surface area, and the mass of the sample, respectively. As a result of the [*X*{(*p*_0_/*p*)} − 1]^−1^ plot against *p*/*p*_0_, the SSA of *α*-Fe_2_O_3_, *γ*-Fe_2_O_3_, and Fe_3_O_4_ are determined to be 4.9, 18.5, and 4.1 m^2^ g^−1^, respectively. It should be noted that the SSA of *γ*-Fe_2_O_3_ is much larger than that of *α*-Fe_2_O_3_ and Fe_3_O_4_ because of the defective structure, as shown in Figure S3b.

In Figure S4, the ^119^Sn-Mössbauer spectra of BaSnO_3_, SnO_2_, and SnO measured from 300 to 20 K were indicated, and the Mössbauer parameters are listed in Tables S4–S6, respectively. Because of the high covalent chemical environment, constant values of *δ* and ∆ ranging from 0.00_±0.01_ to 0.05_±0.01_ mm s^−1^ and from 0.00_±0.01_ to 0.20_±0.01_ mm s^−1^, respectively, were observed from the temperature-dependent ^119^Sn-Mössbauer spectra of BaSnO_3_. In addition, a stable full width at half maximum, *Г*, between 1.23_±0.02_ and 1.18_±0.02_ mm s^−1^ was observed. These results show that the chemical environment of Sn^IV^ in BaSnO_3_ is stable regardless of the temperature.

On the other hand, increasing *δ* values from 0.02 _±0.01_ to 0.08 _±0.01_ mm s^−1^ and stable ∆ of 0.54_±0.01_ mm s^−1^ and *Г* of 0.96_±0.02_ mm s^−1^ were observed from the temperature-dependent ^119^Sn-Mössbauer spectra of SnO_2_. Similarly, temperature-dependent ^119^Sn-Mössbauer spectra of SnO are composed of two doublets. The major component showed increases in absorption area (*A*) from 94.8 to 100%, *δ* from 2.79_±0.01_ to 2.85_±0.01_ mm s^−1^, and *Г* from 0.88_±0.01_ to 1.09_±0.01_ mm s^−1^, while showed the stable ∆ of 1.38_±0.01_ mm s^−1^ with the decrease in temperature.

It is very well known that the BaSnO_3_, SnO_2_, and SnO, respectively, have perovskite, rutile, and tetragonal lead monoxide structures. In BaSnO_3_, the chemical bond of Sn^IV^ -O has high covalency reflected by the small and stable *δ* value between 0.00 and 0.05 mm s^−1^. Together, the Sn^IV^O_6_ unit has high symmetry, which is reflected on the small ∆ value between 0.00 and 0.20 mm s^−1^.

On the other hand, the larger values of *δ* increasing from 0.02 to 0.08 mm s^−1^ and ∆ stable at 0.54 mm s^−1^ compared with BaSnO_3_ were observed for the Mössbauer parameters of SnO_2_, indicating that the chemical bond between the Sn^IV^-O^2−^ becomes more covalent, and the Sn^IV^O_6_ unit becomes more distorted. As compared to the chemical environment of Sn^II^ in SnO with BaSnO_3_ and SnO_2_, the chemical bond between Sn^II^-O becomes significantly ionic because of the larger *δ* between 2.79 and 2.85 mm s^−1^, and the distortion of Sn^II^-O_3_ becomes larger around 1.39 mm s^−1^ because of the unpaired electron on Sn^II^ [43,44].

**Figure 1:**
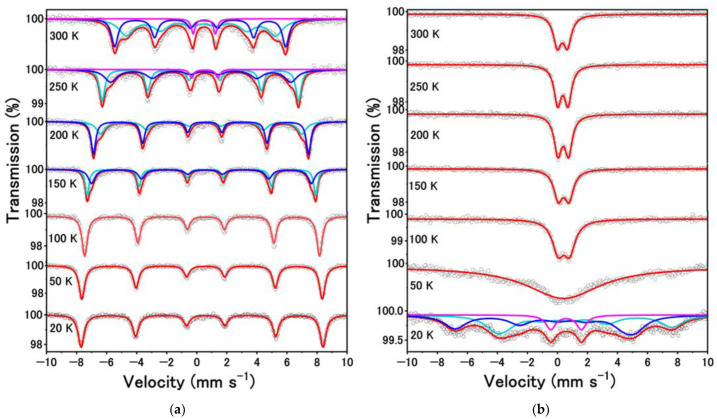

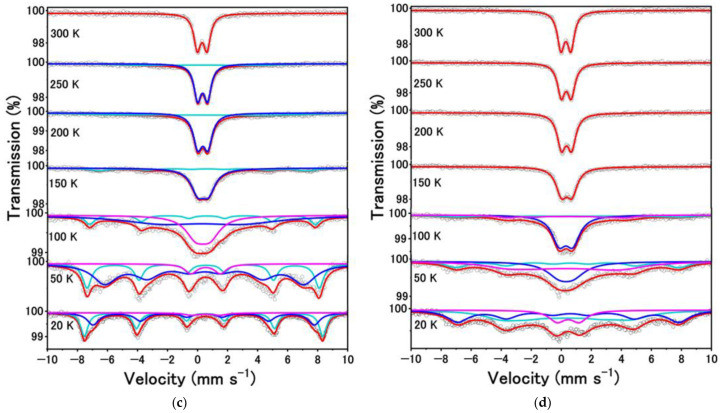
^57^Fe-Mössbauer spectra of *α*-Sn*_x_*Fe_1-*x*_OOH NPs with ‘*x*’ of 0 (**a**), 0.10 (**b**), 0.15 (**c**), and 0.20 (**d**) measured at 300, 250, 200, 150, 100, 50, and 20 K. The subspectra with the different colors in the Figure are matched with the corresponding Mössbauer parameters shown in Table 1, Table 2, Table 3 and Table 4 respectively.

Based on the chemical environment of Fe in *α*-Fe_2_O_3_, *γ*-Fe_2_O_3_, and Fe_3_O_4_, and Sn in BaSnO_3_, SnO_2_, and SnO investigated by ^57^Fe- and ^119^Sn-Mössbauer spectroscopy reported in this paper, a precise structural comparison for *α*-Sn*_x_*Fe_1−*x*_OOH NPs can be performed. ^57^Fe-Mössbauer spectra of *α*-Sn*_x_*Fe_1−*x*_OOH NPs with *x* of 0.0, 0.10, 0.15, and 0.20 measured from 300 K to 20 K are shown in Figure 1, and the corresponding Mössbauer parameters are listed in Table 1, Table 2, Table 3 and Table 4 respectively.

As shown in Figure 1a and Table 1, two sextets with *δ*, ∆, and *H*_int_ of 0.38 mm s^−1^, −0.19 mm s^−1^, and 31.0 T together with 0.37 mm s^−1^, −0.26 mm s^−1^, and 35.4 T due to goethite in addition to a doublet with *δ* and ∆ of 0.49 and 1.47 mm s^−1^ due to superparamagnetic components were observed for ^57^Fe-Mössbauer spectrum of *α*-FeOOH NPs measured at 300 K. These three components were unified into one sextet with *δ*, ∆, and *H*_int_ of 0.46 mm s^−1^, −0.25 mm s^−1^, and 48.7 T when it was measured below 100 K.

As shown in Figure 1b and Table 2, a superparamagnetic doublet due to ferrihydrite with *δ* and ∆ of 0.33 and 0.67 mm s^−1^ was observed from the ^57^Fe-Mössbauer spectrum of *α*-Sn_0.10_Fe_0.90_OOH NPs measured at 300 K, which were increased up to 0.43 mm s^−1^ in *δ* and 0.76 mm s^−1^ in ∆ when the spectrum was measured below 100 K.

The room temperature ^57^Fe-Mössbauer spectrum of *α*-Sn*_x_*Fe_1−*x*_OOH NPs with of ‘*x*’ 0.15 showed a doublet with *δ* and ∆ of 0.31 and 0.64 mm s^−1^. However, two sextets with *δ*, ∆, and *H*_int_ of 0.46 mm s^−1^, −0.12 mm s^−1^, and 49.4 T plus 0.44 mm s^−1^, −0.06 mm s^−1^, and 45.8 T were observed for the ^57^Fe-Mössbauer spectrum of the same sample measured at 20 K. It is recognized that a sextet for the Sn15 appeared in the ^57^Fe-Mössbauer spectrum measured at 250 K, although it can be observed at the lower temperature of 50 K for the ^57^Fe-Mössbauer spectrum of Sn10 NPs, and 100 K for Sn20. It is considered that the size of magnetic ordering is larger in Sn15 NPs than that of Sn10 and Sn20.

**Figure 2:**
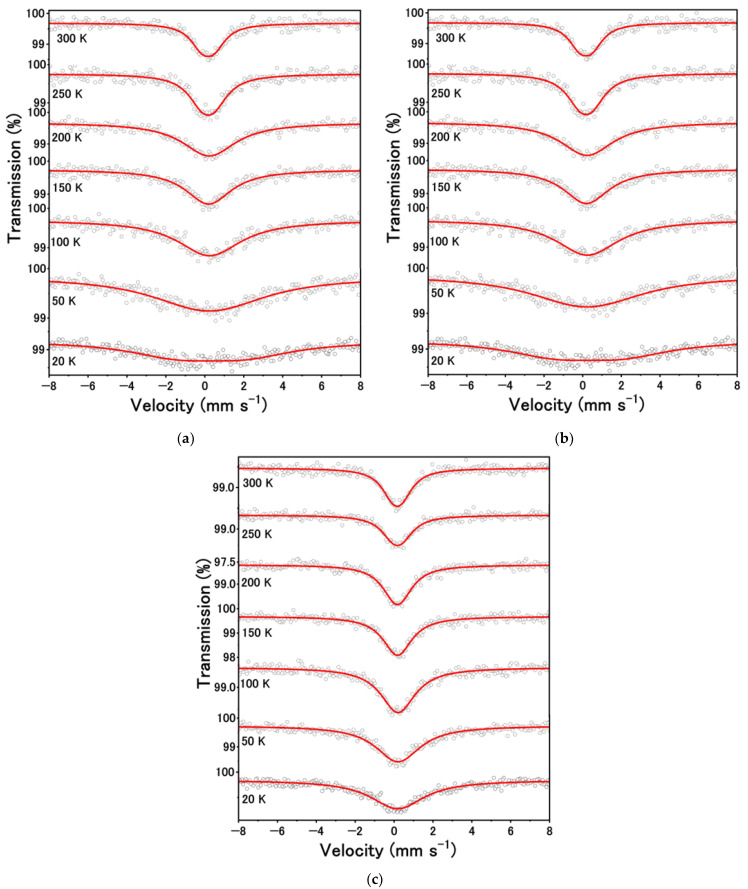
^119^Sn-Mössbauer spectra of *α*-Sn*_x_*Fe_1-*x*_OOH NPs with ‘*x*’ of 0.10 (**a**), 0.15 (**b**), and 0.20 (**c**) measured at 300, 250, 200, 150, 100, 50, and 20 K.

As shown in Figure 2 and Table 5, Table 6 and Table 7, the temperature-dependent ^119^Sn-Mössbauer spectra of α-Sn*_x_*Fe_1−*x*_OOH NPs with ‘*x*’ of 0.10 and 0.20 showed stable *δ* values of 0.09_±0.01_ and 0.10_±0.01_ mm s^−1^, respectively, regardless of the temperature. In contrast, that of 0.15 showed an increase in the *δ* value from 0.09 to 0.24 mm s^−1^ with the temperature decrease from 300 to 20 K, as indicated in Table 6. On the other hand, in almost all sample series, the ∆ value decreased to 0.00 mm s^−1^ when the temperature was decreased from 300 to 20 K for *α*-Sn*_x_*Fe_1−*x*_OOH NPs with of ‘*x*’ 0.10, 0.15, and 0.20.

So far, the temperature-dependent ^57^Fe- and ^119^Sn-Mössbauer spectra were indicated for the iron and tin compounds as reference materials for *α*-Sn*_x_*Fe_1−*x*_OOH NPs with ‘*x*’ of 0.0, 0.10, 0.15, and 0.20 because the authors want to identify the more precise chemical environment of iron and tin in the nanoparticles which is reflected in the physical properties, such as the photocatalytic ability and cathode active properties in secondary batteries [44,45,46].

Generally speaking, the recoilless fraction, *f*, of the Mössbauer nuclei like Fe and Sn can be expressed as follows [47,48]:

(7) $$f=exp\left\{\frac{4\pi^{2}}{\lambda_{\gamma }}<u^{2}>\right\}\;$$

where λ_γ_ and <*u*^2^> are the wavelength of γ-ray energy and mean square displacement, respectively. By using the Debye model, *f* can be converted to the following equation [47,48], i.e.:

(8) $$f=exp\left[-\left(\frac{{3E}_{R}}{2kB\Theta_{D}}\right)\left\{{1+4\left(\frac{T}{\Theta_{D}}\right)}^{2}\cdot \int_{0}^{\Theta /T}\frac{x}{e^{x}-1}\cdot dx\;\right\}\right]$$

where *Θ*_D_, *E*_R_, and *k*_B_ are the Debye temperature, recoil energy, and the Boltzmann constant, respectively. In the sample with a small thickness, the recoilless fraction, *f*, can be approximated by the absorption area, *A*.

In general, the absorption area of the Mössbauer spectra decreases with the temperature. The value of *Θ*_D_ becomes large when the temperature dependence of *f* is small, and it becomes small when *f* is large. Therefore, *Θ*_D_ indicates the rigidness of the surroundings of the Mössbauer nuclei. For example, in oxide glasses, the *Θ*_D_ of Fe^3+^ is larger than 280 K when it acts as a network former (NWF) with high covalency and is smaller than 280 K when it acts as a network modifier (NWM) with high ionicity [46].

**Figure 3:**
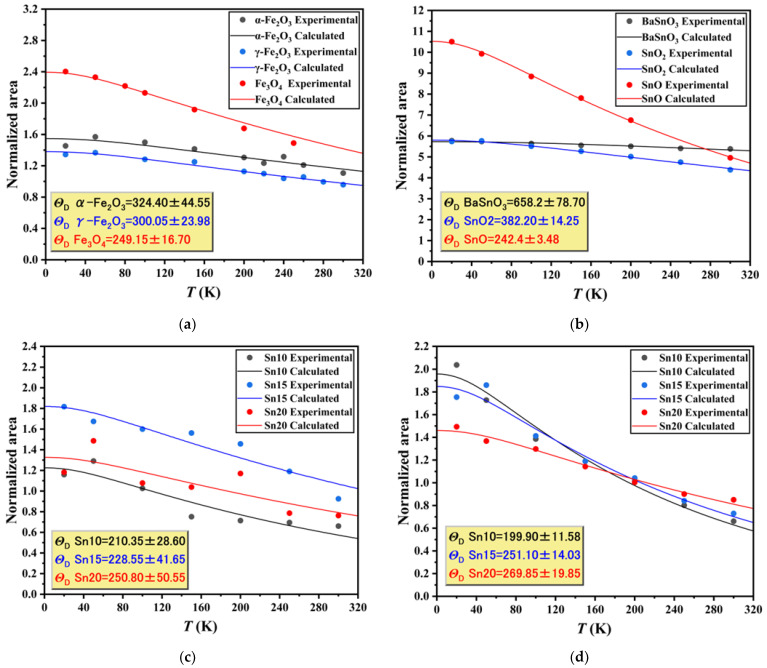
The temperature dependence of the normalized absorption area for (**a**) iron oxide, (**b**) tin compounds, and (**c**) *α*-Sn*_x_*Fe_1-*x*_OOH NPs derived from the ^57^Fe- and (**d**) ^119^Sn-Mössbauer spectra of *α*-Sn*_x_*Fe_1-*x*_OOH NPs with ‘*x*’ of 0.10, 0.15, and 0.20 measured at 300, 250, 200, 150, 100, 50, and 20 K.

As shown in Figure 3 and Figure S5, the absorption area measured at a temperature between 20 and 300 K of ^57^Fe-and ^119^Sn Mössbauer spectra normalized by that measured at 20 K of *α*-Sn*_x_*Fe_1−*x*_OOH NPs were plotted against the temperatures. As references, the same evaluation was conducted for iron compounds of *α*-Fe_2_O_3_, *γ*-Fe_2_O_3_, and Fe_3_O_4_, and tin compounds of BaSnO_3_, SnO_2_, and SnO. 

The temperature dependency of the normalized absorption area (*A*(*T*)/*A*(20 K)) in the ^57^Fe-Mössbauer spectra of iron oxides and *α*-Sn*_x_*Fe_1−*x*_OOH NPs are shown in Figure 3a,c. The *f* value decreased from 100% to 76, 71, and 61% for *α*-Fe_2_O_3_, *γ*-Fe_2_O_3_, and Fe_3_O_4_ when the ^57^Fe-Mössbauer spectra were measured from 20 to 300 K, as shown in Figure S5. By using Equation (8), the *Θ*_D_ values were determined at 324, 300, and 249 K, respectively. It can be understood that the largest *Θ*_D_ value of 324 K of *α*-Fe_2_O_3_ is the largest because of the large coordination number of 6 and that the smaller *Θ*_D_ value of 300 K for *γ*-Fe_2_O_3_ was obtained because of the smaller coordination number of 4 with the defective structure. Further, the smaller *Θ*_D_ of 249 K was obtained for Fe_3_O_4_ because it contains a mixed valence state of Fe^2+^ and Fe^3+^ with the vacant sites [45,46].

On the other hand, the decrease in *f* values were estimated from 100% to 76, 57, 58, and 53% for α-Sn*_x_*Fe_1−*x*_OOH NPs with ‘*x*’ of 0.10, 0.15 and 0.20, respectively, of which *Θ*_D_ values were determined by 210, 228, and 250 K. It can be recognized that the *Θ*_D_ values of tin-containing *α*-Sn*_x_*Fe_1−*x*_OOH NPs become smaller than that of α-Fe_2_O_3_ (=324 K) and comparable for γ-Fe_2_O_3_ (=300 K) and Fe_3_O_4_(=249 K). The *Θ*_D_ of Sn10, Sn15, and Sn20 are gradually increased with the increasing Sn content.

In addition, the obtained *Θ*_D_ values of *α*-Sn*_x_*Fe_1−*x*_OOH NPs are comparable to those of NWM in oxide glasses. As shown in Figure 3b, the *A*(*T* K)/*A*(20 K) value of the temperature-dependent ^119^Sn-Mossbauer spectra of BaSnO_3_, SnO_2_, and SnO decreased from 100% to 93, 76, and 47%, of which the *Θ*_D_ values were estimated to be 658, 382, and 242 K, respectively. It is noted that the *Θ*_D_ value of Sn^IV^ in BaSnO_3_ is extremely large because the *f* value is consistent between temperatures of 20 and 300 K. Significantly small values of *A*(*T* K)/*A*(20 K) at 300 K and *Θ*_D_ values were evaluated for 32% and 199 K, 41% and 251 K, and 57% and 269 K for *α*-Sn*_x_*Fe_1−*x*_OOH NPs with ‘*x*’ of 0.10, 0.15, and 0.20, respectively. It is interesting to recognize that the *Θ*_D_ value of Sn^IV^ in *α*-Sn*_x_*Fe_1−*x*_OOH NPs is smaller than that of BaSn^IV^O_3_ (=658 K) and Sn^IV^O_2_ (=382 K) and is comparable to that of Sn^II^O (=242 K). These results indicate that the surrounding of Sn^IV^ in α-Sn*_x_*Fe_1−*x*_OOH NPs is loosely bound in the oxide matrix because of its nanostructure, i.e., the short distance of atomic ordering [47]. In addition, the *Θ*_D_ values of Sn10, Sn15, and Sn20 using ^57^Fe- and ^119^Sn-Mössbauer spectra are in good correlation with TEM and BET analysis [16], where the sample with higher SSA (Sn10) has a lower value of *Θ*_D_, while with the decrease in SSA, i.e., the increase in crystal size, the Debye temperature is higher [48].

These unique nanostructures characterized by temperature-dependent ^57^Fe- and ^119^Sn-Mössbauer spectra reflect on the electrical conductivity and cathode active ability in secondary batteries.

**Figure 4:**
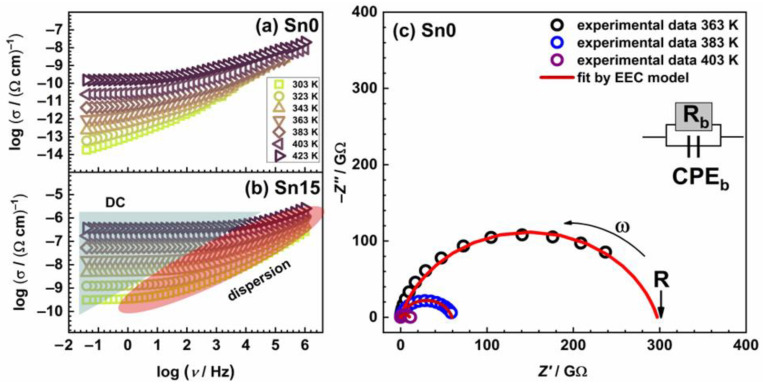
Conductivity spectra for (**a**) Sn0, (**b**) Sn15 from *α*-Sn*_x_*Fe_1−*x*_OOH NPs series, and (**c**) complex impedance plane (Nyquist plot) and spectra for Sn0 @various temperatures with corresponding equivalent circuit used for fitting the data. Empty symbols represent the experimental data, while the lines depict the fit obtained through EEC modelling.

Experimental data obtained through solid-state impedance spectroscopy (SS-IS) for *α*-Sn*_x_*Fe_1−*x*_OOH NPs are presented as conductivity spectra measured in a wide temperature and frequency range. The conductivity spectra for Sn0 and Sn15 samples, as representative samples from this work, are shown in Figure 4a,b.

Although the conductivity isotherms are similar in shape for different samples and at different temperatures, overall, two different spectral features can be identified. The first one is a nearly frequency-independent conductivity related to the long-range transport of charge carriers which corresponds to DC conductivity or the overall resistance and can be observed at low frequencies. The second one is the frequency-dependent part, commonly referred to as conductivity dispersion, which occurs with increasing frequency in the form of a power-law and is related to localized short-range motions of charge carriers. One can see that the change from DC to the dispersion region shifts with an increase in temperature to higher frequencies (Figure 4a,b). For the lower-conducting sample, Sn0, DC plateau is not observed at lower temperatures, due to our experimental frequency window, and the conductivity is strongly frequency-dependent.

As a step forward, we examined the data in a complex impedance plane, known as the Nyquist plot. The impedance spectra at different temperatures for Sn0 NP and their corresponding electrical equivalent circuit (EEC) are shown in Figure 4c. For all studied α-Sn*_x_*Fe_1−*x*_OOH NPs, the impedance spectrum contains a single semicircle related to the bulk effects within the studied samples. The analysis of these plots involves EEC modelling with a complex nonlinear least-square (CNLLSQ) fitting procedure. Typically, a single impedance semicircle is represented by a simple EEC model with a resistor and a capacitor connected in parallel. Ideally, semicircles pass through the origin of a plot and yield a low-frequency intercept on the real axis, corresponding to the resistance of the observed process. However, usually semicircle is depressed, and the constant-phase element (CPE) is used instead of the standard capacitor in the equivalent circuits. The CPE is an empirical impedance function of the type: *Z*_CPE_ = 1/*A*(*iω*)*^α^*, where *A* and *α* are the constants. The parameters for each circuit element (*R*, *A*, and *α*) were determined directly from the measured impedance data using the CNLLSQ method. By modelling data, taking into account the parameters obtained and the sample geometry (see Section 3), we calculated the DC values, as shown in Table 8. A good correlation is observed between these values and the DC conductivity read from the conductivity spectra, see Figure 4a,b and the DC plateau at each temperature.

In our case, one can see that impedance spectra have similar shapes but are not, however, fully defined at lower temperatures, see Figure 4c. The radius of the semicircle decreases with the increasing temperature indicating that electrical transport is thermally activated, and semiconducting behavior is observed. In contrast, after an increase in temperature, a full semicircle is present. This effect is also visible in the conductivity spectra, Figure 4a,b, where at lower temperatures the DC plateau is not observed due to the experimental setup and frequency window used in this study. In such cases, the modelling data approach was shown to be useful in determining DC conductivity values.

**Figure 5:**
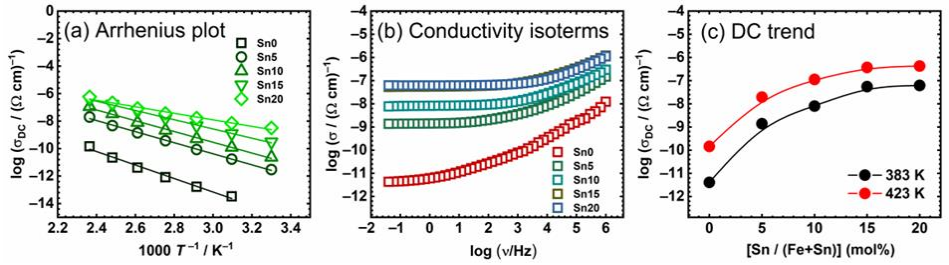
(**a**) Arrhenius plots of DC conductivity and (**b**) conductivity isotherms measured @383 K for individual samples from *α*-Sn*_x_*Fe_1−*x*_OOH NPs series; (**c**) compositional dependence of DC conductivity at various temperatures for individual *α*-Sn*_x_*Fe_1−*x*_OOH NPs. Lines in (**a**) are obtained through linear regression, while the lines connecting data points in (**c**) are drawn as guides for the eye.

The DC conductivity exhibited by our samples manifests a temperature-dependent behavior conforming to an Arrhenius relationship, indicative of semiconducting characteristics, see Figure 5a. Consequently, the activation energy for DC conductivity, *E*_DC_, was determined for individual samples from the slope of log(*σ*_DC_) versus 1000/T using the relation: $\sigma_{\mathrm{DC}}T\;=\sigma_{0}^{*}\exp\left(\frac{\text{–}E_{\mathrm{DC}}}{k_{B}T}\right)$, where *σ*_DC_ is the DC conductivity, $\sigma_{0}^{*}\;$ is the pre-exponent, k_B_ is the Boltzmann constant, and *T* is the temperature (K). The calculated activation energy spans a wide range, varying from ~94 kJ/mol to ~45 kJ/mol, contingent on the composition as the tin content increases up to *x* = 0.2.

Upon sample comparisons within the series of *α*-Sn*_x_*Fe_1−*x*_OOH NPs, intriguing insights emerge concerning the evolution of the conductivity isotherm with the modification of NP as the tin content increases. Conductivity isotherms measured @383 K for individual samples are depicted in Figure 5b. Notably, the overall shape of the isotherms remains unaltered across the observed features, suggesting an unaffected mechanism of electrical transport. As the Sn content increases, a discernible shift from DC to the dispersion region is observed at higher frequencies, similar to the temperature effect (Figure 4a,b) and the DC plateau extends over a wider frequency range, which is characteristic of fast electron transport [49,50]. Simultaneously, the Nyquist plot shown in Figure 4c reveals the presence of only one semicircle without additional signatures, such as a second semicircle indicative of an additional contribution to overall DC conductivity or a spur at lower frequencies, a signature of ionic transport.

Going back to DC conductivity, compositional dependence at different temperatures for individual *α*-Sn*_x_*Fe_1−*x*_OOH NPs is shown in Figure 5c. Modification of NPs and the increase in Sn content has a positive effect, resulting in an increase in DC conductivity for almost 5 orders of magnitude, see Figure 5c and Table 8. Interestingly, as the Sn/(Fe + Sn) ratio increases, the DC conductivity exhibits a non-linear trend, from starting sample without Sn (Sn0) with *σ*_DC_ value of 9.37 × 10^−7^ (Ω cm)^−1^ @150 °C, up to DC plateau for samples containing 0.15 and 0.20 Sn (Sn15 and Sn20) with a DC value of ~4 × 10^−7^ (Ω cm)^−1^ @423 K. This non-linear conductivity pattern and levelling at a higher Sn content suggests that structural modifications have a notable impact on electron transport, and this effect could not be solely attributed to a linear decrease in Fe concentration within the studied samples. At the same time, *E*_DC_ follows the opposite trend. In goethite, charge transport is primarily governed by the thermally activated via three-dimensional hopping of small polarons (SPH), with various inequivalent paths for electron hopping, mediated by O and OH species, across the lattice. In our recent study on *α*-Ni_x_Fe_1−x_OOH NPs [17], the obtained values for the activation energy (0.75–0.85 eV) are lower in comparison to the goethite studied by Guskos et al. [18]. In this work on *α*-Sn*_x_*Fe_1−*x*_OOH NPs, with a narrow composition, up to *x* = 0.2, we observed a similar range in comparison to activation energies for Ni*_x_*Fe_1−*x*_OOH NPs [17]. In both our studies, the observed values align more closely with the values reported for magnetite and hematite. Moreover, this range of values we observed is in good correlation with the data in the literature on various materials with dominant electron transport and (partially) disordered structure [49,50]. Interestingly, the presence of mixed protonic–electronic conduction in goethite was identified by Su et al. [51] in the study on electrical properties under pressure by impedance spectroscopy. It shows how different conditions can influence the overall transport mechanism.

In this work, the augmentation of Sn content results in an increase in DC conductivity, an effect likely attributed to alterations in bonding and modifications in the structure. The observed feature could be rationalized with the introduction of tin into goethite, the Sn^IV^-O chemical bond becomes shorter, and the chemical bond Fe^III^-O is longer, confirmed by Mössbauer [16]. These results indicate that an electron on bridging oxygen (O^2−^) between Fe^III^ and Sn^IV^ is strongly attracted to Sn^IV^, which causes a decrease in the bandgap energy of goethite. This reduction, in turn, fosters the establishment of continuous conduction pathways, facilitating uninterrupted electrical transport. The probability of jumping for electrons increases, as a result of the decrease in barrier magnitude impeding electron movement. Furthermore, the presence of mixed metal centers and the potential formation of local Fe-Sn pairs, which play a role as donor centers, contribute to the generation of electrons and consequently affect the conductivity [52].

The most visible increase in DC conductivity values is conspicuous when introducing *x* = 0.05 Sn in the goethite structure, exhibiting a three-order-of-magnitude increase. With additional tin introduction, the conductivity continues to rise, albeit at a slower rate, eventually reaching a plateau at the highest doped tin concentrations, 0.15–0.20. In general, the electrical conductivity mechanism in investigated samples seems to be related to a combination of two dominant factors: (i) ease of electron/polaron mobility jump along with (ii) a decrease in charge carrier concentration. In our previous study [17], the introduction of Ni at higher concentrations (*x* = 0.1–0.5) resulted in a decrease in conductivity throughout the series, albeit higher than the values for pure goethite up to 0.5 Ni-doped goethite. On the other hand, in this study, we introduced up to *x* = 0.2 of Sn, with a positive effect and increase in conductivity. The observed plateau at highest Sn concentration indicates that the decrease in charge carriers overtakes the overall trend, and cannot be compensated with the observed ease of polaron jumps above *x* > 0.15 Sn NP. Additional Sn introduction beyond this point is anticipated to lead to a decrease in conductivity. Additionally, the observed DC trend is in good correlation with TEM and BET analysis [16], where samples with lower SSA and needle-shaped samples (Sn0 and Sn5) have lower values of electrical conductivity, while with an increase in SSA, i.e., decrease in crystal size, electrical conductivity is higher. Based on the above, the Sn15 sample emerges as an optimal composition.

Overall, all of the above-mentioned coupled with structural features play a crucial role in the increase in DC conductivity and overall electrical transport with doping of goethite with tin up to 0.20 in the *α*-Sn*_x_*Fe_1-_*_x_*OOH nanoparticles from this study.

To evaluate the *α*-Sn*_x_*Fe_1−*x*_OOH NPs as cathode materials for both sodium-ion (Na-ion) and lithium-ion (Li-ion) batteries, charge and discharge capacities were measured at the low current rate of 5 mA g^−1^ and the high current rate at 50 mA g^−1^ in the wide voltage range from 0.8 to 4 V.

**Figure 6:**
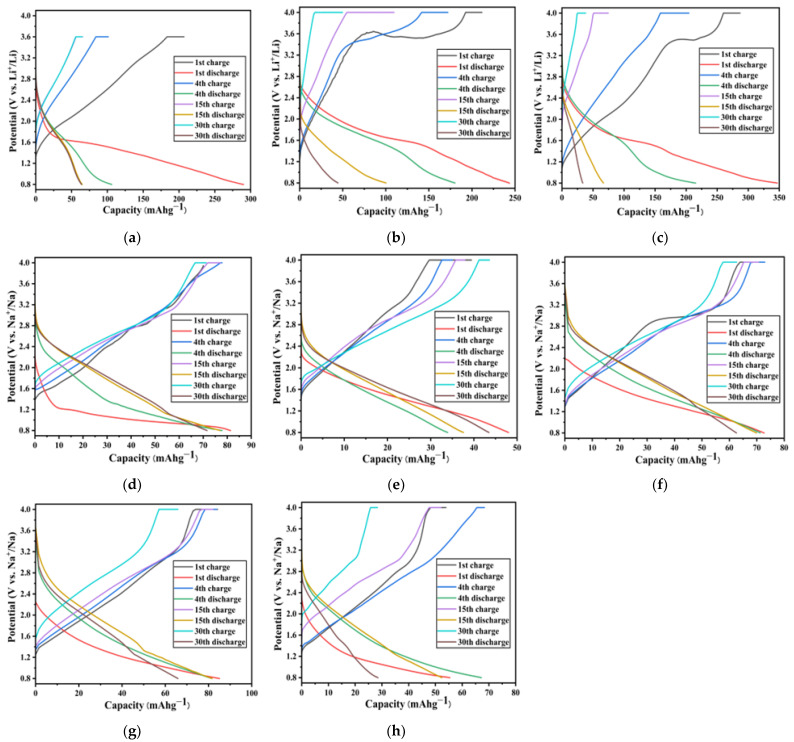
Charge–discharge curves of Li-ion battery (**a**) Sn0, (**b**) Sn10, and (**c**) Sn15 and Na-ion battery (**d**) Sn0, (**e**) Sn5, (**f**) Sn10, (**g**) Sn15, and (**h**) Sn20 as cathode recorded under current density of 50 mA g^−1^.

The voltage versus capacity profiles during the charge and discharge processes of *α*-Sn*_x_*Fe_1−*x*_OOH NPs at the high current rate of 50 mA g^−1^ were analyzed at the 1st, 4th, 15th, and 30th cycles for both Na- and Li- ions batteries, as illustrated in Figure 6.

Figure 6a–c show the charge–discharge capacities of Sn0, Sn10, and Sn15, respectively, as cathode materials in LIBs. A small plateau at 1.61 V was observed in the first discharge in Sn0, Sn10, and Sn15, corresponding to Li intercalation before the reduction reaction, given the layered structure of α-FeOOH [13,49,52,53,54,55,56]. In the first charge cycle of Sn0, the voltage gradually increases to 3.6 V without a significant plateau, while Sn10 and Sn15 exhibited plateaus at 3.56 and 3.45 V and the voltage increased to 4 V. In addition, the large irreversible capacity shown in Sn0 can be attributed to the formation of LiOH and/or decomposition of a polytetrafluoroethylene (PTFE) binder that occurred during the discharge. The Sn substation in the *α*-FeOOH NPs showed a positive effect in the voltage range. Inherent conversion reactions are a notable limitation attributable to their polarization, characterized by a substantial divergence in voltage during charge and discharge cycles [57,58]. For instance, transition metal oxides exhibit a voltage separation of approximately 0.9 V. The significant hysteresis observed in Figure 6, depicting the disparity between reduction and oxidation reactions, is likely attributed to the inadequate conductivity, encompassing both the electronic and ionic facets inherent in the compound [52,53].

The initial discharge capacities of Sn0, Sn10, and Sn15 were 290, 243, and 347 mAh g^−1^ during the insertion, and the reversible capacity was 206, 212, and 287 mAh g^−1^, respectively. However, the discharge capacity of all samples decreased with an increasing number of cycles, dropping to 63.8, 43.7, and 35.9 mAh g^−1^.

On the other hand, the charge–discharge curves of Sn0, Sn5, Sn10, Sn15, and Sn20 NPs were presented in Figure 6d–h as cathode materials in SIB. The initial discharge capacities of Sn0, Sn5, Sn10, Sn15, and Sn20 were 81, 43, 73, 85, and 55 mAh g^−1^, respectively. The first discharge process of Sn0 exhibited a long flat plateau around 1.13 V referring to the reactions of FeOOH with Na^+^ ions according to the following equation: *α*-FeOOH + Na^+^ + e^−^ → FeO + NaOH. Additionally, a small plateau at 1.25 V was observed in the first discharge of Sn0 NPs corresponding to sodium intercalation before the reduction reaction. In contrast, the first charge process of Sn0 displayed two small plateaus at 2.74 V and 3.25 V attributed to FeO + NaOH → *α*-FeOOH+ Na^+^ + e^−^ [52,58]. After Sn substitution in *α*-FeOOH, in the first discharge process, the long flat plateau observed in Sn0 around 1.13 V disappeared in all samples. During the charging process, no significant changes were observed in Sn5 NPs sample. However, starting from Sn10, a long flat plateau at 3.1 V appeared, while Sn15 NPs and Sn20 NPs showed a small plateau at 2.85 and 3.15 V, respectively.

**Figure 7:**
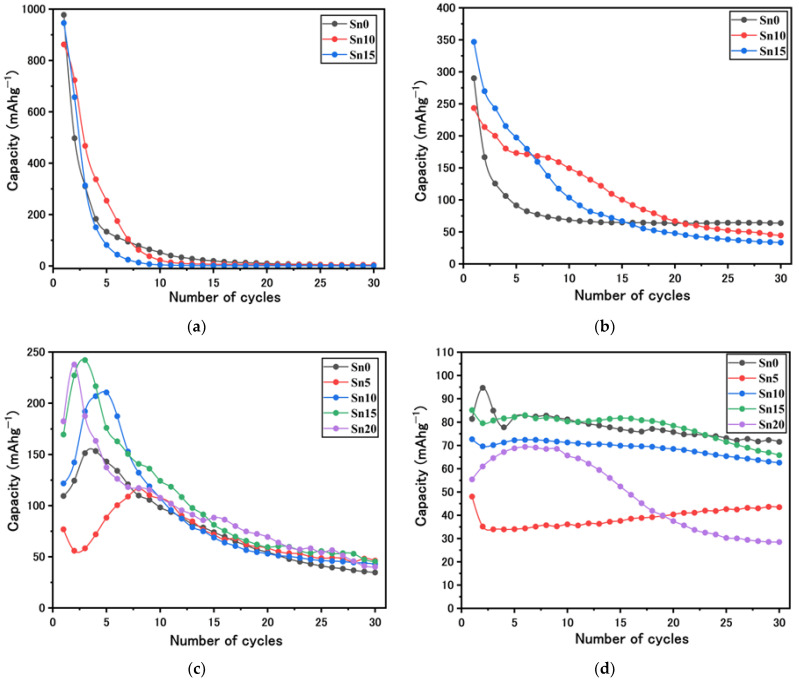
Cyclability of Li-ion battery (LIB) and Na-ion battery (SIB) containing *α*-Sn*_x_*Fe_1−*x*_OOH NPs cathode recorded under current density of 5 mA g^−1^ and 50 mA g^−1^, (**a**) LIB at 5 mA g^−1^, (**b**) LIB at 50 mA g^−1^, (**c**) SIB at 5 mA g^−1^, and (**d**) SIB at 50 mA g^−1^.

Figure 7 and Table 9 present the discharge capacity and capacity retention of *α*-Sn*_x_*Fe_1−*x*_OOH NPs as cathode materials for both lithium-ion batteries (LIBs) and sodium-ion batteries (SIBs), measured at low (5 mA g^−1^) and high (50 mA g^−1^) current rates across a wide voltage range from 0.8 to 4.0 V for 30 cycles.

In the context of LIBs, Figure 7a illustrates the initial capacity at a low current rate of 5 mA g^−1^ for Sn0, Sn10, and Sn15, which is notably high at 977, 862, and 945 mAh g^−1^, respectively. The capacity retention (%) after 30 cycles is reported as 0.4, 0.5, and 0.1%, respectively. Figure 7b depicts the initial capacities of Sn0, Sn10, and Sn15 at a high current rate of 50 mA g^−1^, measured at 290, 243, and 347 mAh g^−1^, with corresponding capacity retention (%) after 30 cycles of 22, 18, and 9.8%, respectively. Notably, the capacity retention measured at the low current rate is considerably smaller than that at the high current rate. It is noteworthy that the substitution of Sn in FeOOH NPs demonstrates a positive impact on the initial capacity, especially with *x* = 0.15, while exhibiting a negative effect on capacity retention, as indicated in Table 9.

Turning to SIBs, Figure 7c displays the initial capacity at a low current rate of 5 mA g^−1^ for Sn0, Sn5, Sn10, Sn15, and Sn20, measured at 110, 77, 122, 170, and 182 mAh g^−1^, with capacity retention (%) after 30 cycles reported as 32, 60, 35, 27, and 22%, respectively. Figure 7d shows the initial capacity at a high current rate of 50 mA g^−1^ for Sn0, Sn5, Sn10, Sn15, and Sn20, measured at 81, 43, 73, 85, and 55 mAh g^−1^, with corresponding capacity retention (%) after 30 cycles of 88, 90, 86, 77, and 51%, respectively. Remarkably, the capacity retention (%) of *α*-Sn*_x_*Fe_1−*x*_OOH NPs significantly increases after performing at a high current rate.

Considering the results for α-Sn*_x_*Fe_1−*x*_OOH NPs as cathode materials in both LIBs and SIBs, particularly focusing on capacity retention after 30 cycles, it can be inferred that the performance of α-Sn*_x_*Fe_1−*x*_OOH NPs as active cathode materials is more comparable in an SIB compared to an LIB.

**Figure 8:**
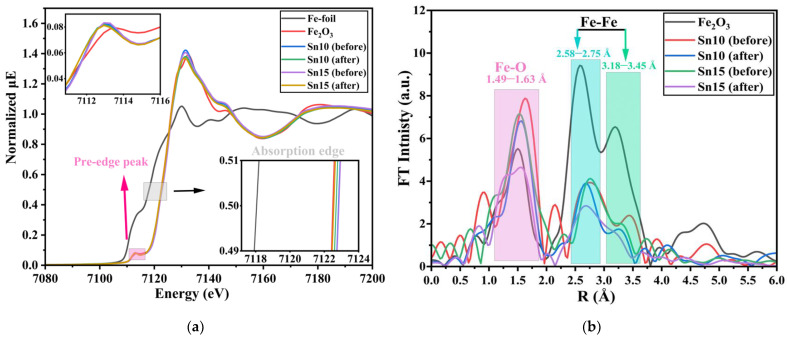
Fe-*K* edge XANES and EXAFS spectra of *α*-Sn*_x_*Fe_1−*x*_OOH NPs cathode with ‘*x*’ 0.10 and 0.15 in a Na-ion battery before and after charge–discharge processes of 30 times, (**a**) XANES and (**b**) EXAFS.

Figure 8 shows the Fe-*K* edge XANSES and Fourier transform of Fe-*K* EXAFS spectra (FT-EXAFS) of Sn10 and Sn15 NPs as prepared samples and after testing the battery for 30 cycles together with standard materials of Fe_2_O_3_ and Fe-foil. 

Figure 8a shows the XANSES spectra in the energy range from 7080 eV to 7200 eV to obtain the changes in pre-edge peak and absorption energy of the S10 and Sn15 compared with the standard materials (Fe_2_O_3_). The absorption energy is defined as the normalized absorbance at 0.5 of each spectrum [54,55]. In addition, when the absorption energy shifts to high, it indicates that the oxidation state of Fe has increased [16,17].

As we can see, the absorption energy of both samples before and after testing the battery and Fe_2_O_3_ are close to each other at approximately 7122.45 eV, therefore, we figured out the part of the spectra and inserted it to the right and at the bottom of Figure 8a. The absorption edge of Sn10 and Sn15 as prepared samples and after testing the battery for 30 cycles are very close together with Fe_2_O_3_, indicating no significant change in the oxidation state of Fe and it is Fe^3+^ state.

The pre-edge peak of Sn10 and Sn15 before and after testing the battery together with Fe_2_O_3_ showed at 7122.73 eV with low peak intensity due to the forbidden electron–dipole transition between 1*s* and 3*d* orbitals [16,58]. Additionally, there are no significant changes in the pre-edge peak in centra and intensity, indicating that the no significant changes in the oxidation state and coordination number of Fe. We can conclude that in Sn10 and Sn15 before and after testing battery Fe exhibited in Fe^3+^ (*T*_d_).

Figure 8b shows the FT-EXAFS of Fe_2_O_3_, Sn10, and Sn15 before and after testing the battery. The first peak observed between 1.49 and 1.63 Å, which is attributed to Fe-O [16,59]. The second and third peaks were observed between (2.58–2.75) Å and (3.18–3.45) Å, both were attributed to Fe-Fe [17].

## 3. Materials and Methods

### 3.1. Preparation 

#### 3.1.1. α-Sn_x_Fe_1-x_OOH NPs 

**Scheme 1:**
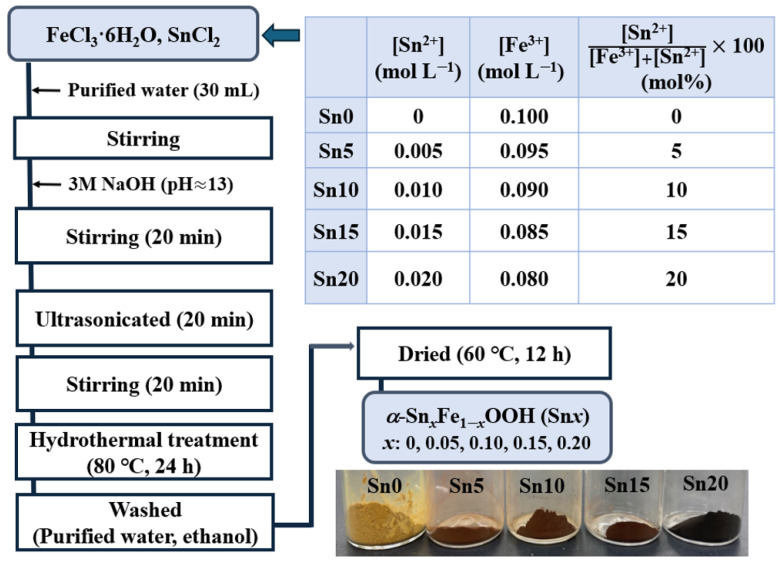
Preparation procedure of *α*-Sn*_x_*Fe_1−*x*_OOH NPs.

The preparation procedure of *α*-Sn*_x_*Fe_1−*x*_OOH NPs by hydrothermal reaction is shown in Scheme 1.

The same protocol was explained elsewhere [16]. In short, starting materials of FeCl_3_•6H_2_O (Wako, Osaka, Japan; 095-00875) and SnCl_2_ (Wako, Osaka, Japan; 204-11491) of the molar ratio of Fe: Sn = 1.00:00, 0.95:0.05, 0.90:0.10, 0.85:0.15, and 0.80:20 were mixed in 30 mL miliQ water for 20 min [16]. A total of 5 milliliters of 3M NaOH aqueous solution was added to the clear solution by droplets to reach the pH of 13 [16]. The obtained suspended solution was additionally mixed for 20 min twice [16]. Between the mixings, ultrasonication was performed for 20 min [16].

The resulting suspended solution was placed into the hydrothermal reactor (Model 4744; Parr, Moline, IL, USA) and reacted at 80 °C for 24 h [16]. The precursor was washed with distilled water and ethanol three times. Finally, the resulting powder of *α*-Sn*_x_*Fe_1−*x*_OOH NPs was obtained after drying the wet products at 60 °C for 12 h [16].

#### 3.1.2. Li- and Na-Ion Battery Containing α-Sn_x_Fe_1−x_OOH NPs Cathode

**Scheme 2:**
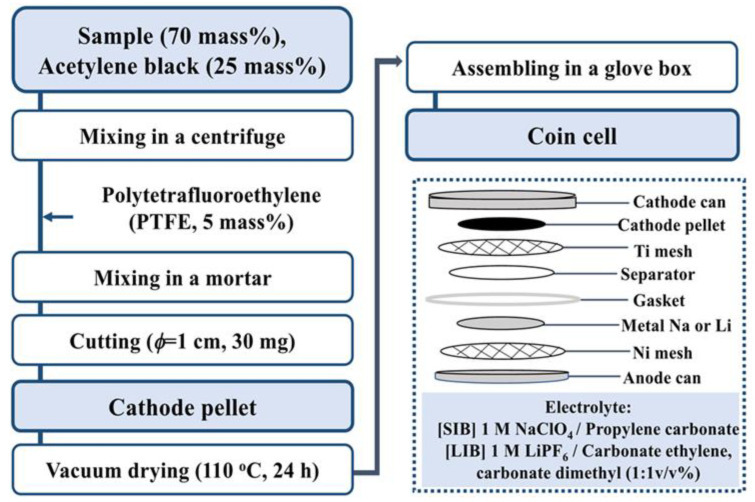
Structure of Na-ion battery.

The Na-ion battery containing the *α*-Sn*_x_*Fe_1−*x*_OOH NPs cathode was assembled with a CR2032 type coin cell in a glove box under the inert gas atmosphere with an O_2_ concentration of less than 1.00 ppm. The preparation procedure is shown in Scheme 2.

To prepare the cathode, 125 mg of *α*-Sn*_x_*Fe_1−*x*_OOH NPs and 45 mg acetylene black (AB, Strem Chemicals 06-0025; Newburyport, MA, USA) were mixed under 800 rpm for 20 min using a Planet M2-3F (Nagao System, Kanagawa, Japan). Then, 95 mg of the ball-milled mixture mixed with 5 mg polytetrafluoroethylene (PTFE; Wako, Osaka, Japan; 165-13412) were mixed to make a pellet. The pellet of the cathode was formed with a 10 mm diameter in 30 mg weight, the mass ratio of *α*-Sn*_x_*Fe_1−*x*_OOH NPs to AB to PTFE as 70:25:5. Ti-mesh fixed the cathode, and Ni-mesh fixed the Na anode (Kishida, Osaka, Japan; 620-70852), with a weight of 90 mg. Each electrode was pressed by applying more than 20 kN for the good contact. In assembling the coin cell, the prepared cathode and anode were separated by a separator (Celgard^®^#3501; Charlotte, NC, USA) and sealed after filling the electrolyte of 1 M NaClO_4_/propylene carbonate (PC) (Tomypure LIPASTE-P/S1; Tokyo, Japan) in the coin cell. As for the Li-ion battery preparation, the same procedure was applied except for using an anode of metallic Li (Kanto-Kagaku, Tokyo, Japan; 24243-35) with 30 mg weight and an electrolyte of 1M/LiPF_6_ ethylene carbonate (EC): dimethylcarbonate (DMC) (1:1 v:v%) (Kishida, Osaka; LBG-00022).

### 3.2. Characterization

#### 3.2.1. XAFS Spectroscopy

The X-ray absorption fine structure (XANES/EXAFS) around the Fe *K*-edge was measured under the transmission mode at the BL-12C or BL-9A at the High Energy Accelerator Research Organization (KEK-PF, 1-1 Oh-ho, Tsukuba, Ibaraki 305-0801, Japan). The specimen for each measurement was formed into a pellet with an 8 mm diameter by pressing at 5 kN the mixture of sample and boron nitride (Wako, Osaka, Japan; 028-02281) with the appropriate ratio for having the sufficient intensity of XANES and EXAFS spectra, which depends on the X-ray energy for the targeting elements. The high-energy X-ray from the synchrotron was monochromatized by adjusting the angle between the double crystals of Si(111) phases. The Ni-mirror filtered out the higher harmonic waves. The specimen was placed between the front and rear gas ionization chambers. The former was filled with N_2_ gas, while the rear was filled with a mixture of N_2_ and Ar gases with a 70 and 30% ratio. Before reaching the detector, the high-energy X-ray went through the front gas ionization chamber, specimen, and rear gas ionization chamber. The detected X-ray intensity was monitored with Quickscan XAFS, and the obtained spectra were analyzed by Athena software version 0.9.26.

#### 3.2.2. Mössbauer Spectroscopy

^57^Fe- and ^119^Sn-Mössbauer spectra were measured with a conventional acceleration method using ^57^Co (Rh) (Ritverc, St. Petersburg, Russia; 1.85 GBq, MCo7.124/74.20; produced on 12 February 2020) and Ca^119^SnO_3_ (Ritverc, Russia; 185 MBq, MSn9.222/43.21; produced on 11 March 2021) as the sources, and *α*-Fe and BaSnO_3_ as the references for the isomer shift, respectively. Samples with a 40 mg weight were homogeneously dispersed in a circular holder with a 10 mm diameter and inserted into the spectrometer. The γ-ray source was connected to the transducer (MVT-1000; Wissel, Kalkar, Germany), which was connected to the driving unit (Wissel, Germany; MDU-1200). The movement of the source was controlled by a digital function generator (Wissel, Germany; DFG-1200). The γ-rays through samples were detected by the proportional counter (45431; Niki-Kogei Inc., Tokyo, Japan), which was charged at 2 kV by the high voltage supplier (556; ORTEC, Atlanta, GA, USA), and the signal was amplified with an amplifier (570; ORTEC, USA) via the preamplifier (142; ORTEC, USA). The amplified signals were monitored with a multi-channel scaler (EASY-MCS; ORTEC, USA) on a Windows PC, and the recorded spectra were analyzed by Mosswinn 4.0i. For the low-temperature Mössbauer measurements, the sample temperature was changed from 20 to 300 K by a cooling system composed of the compressor (CW303; ULVAC CRYOGENICS Inc., Kanagawa, Japan) and the coldhead (M310; ULVAC CRYOGENICS Inc., Kanagawa, Japan), in which the sample chamber was filled with He gas and a high vacuum was maintained of less than 1 Pa achieved by a turbo-pump (Hi-cube 80 Eco; Pfeiffer Vacuum Gmbh, Aßlar, Germany). The Debye temperatures derived from temperature-dependent ^57^Fe- and ^119^Sn-Mössbauer spectra of *α*-Sn*_x_*Fe_1−*x*_OOH NPs were calculated using the Debye Fit 1.0 developed by Dr. Prof. Istvan Virág.

#### 3.2.3. BET Surface Area Analysis

The Brunauer–Emmett–Teller (BET) method was applied for the evaluation of the specific surface area (SSA) using the BERSORP MINI X (MICROTRAC BEL, Osaka, Japan). N_2_ gas was flown as the adsorbate. The obtained results were analyzed by BELMaster7 (MICROTRAC BEL).

#### 3.2.4. Electrical Impedance Spectroscopy

A comprehensive study of the electrical properties of the prepared samples was conducted through solid-state impedance spectroscopy (SS-IS). Polycrystalline specimens were pressed into cylindrical pellets (diameter of 10 mm, thickness ~0.5 mm) under a uniform load of 2 tons using a hydraulic press. For the establishment of electrical contacts and subsequent measurements in cross-section setup, gold electrodes were deposited on the top and bottom surface of the disk-shaped samples using the Sputter Coater SC7620 (Quorum Technologies, Lewes, UK). The Novocontrol Alpha-AN-Dielectric Spectrometer (Novocontrol Technologies GmbH & Co. KG, Montabaur, Germany) was employed across a broad frequency range spanning from 0.04 Hz to 1 MHz to gather complex impedance data. The measurements were conducted at temperatures ranging from 30 °C to 150 °C, with a controlled increment of 20 °C and temperature accuracy within ±0.2 °C. Heating/cooling was controlled with LN2, which means an inert atmosphere is present. Electrical equivalent circuit (EEC) modeling was applied on obtained experimental data using the complex nonlinear least-squares (CNLLSQ) fitting procedure and WinFIT software (version 3.2; Novocontrol Technologies GmbH & Co. KG, Hundsangen, Germany). Parameters derived from the fitting procedure, including resistance (*R*), along with electrode dimensions (sample thickness, *t*, and electrode area, *A*), were utilized to calculate the DC conductivity, σ_DC_ = *t*/(*R* × *A*).

#### 3.2.5. Li- and Na-Ion Battery Performances

The performances of the Na- and Li-ion batteries were monitored in the charge–discharge process, repeated up to 30 times using TOSCAT 3100SK (Toyo-System, Fukushima, Japan) under the setting voltage from 0.8 to 4.0 V with the current density of 5 and 50 mA g^−1^.

## 4. Conclusions

The relationship among the structure, electrical conductivity, and the cathode performances in the Li- and Na-batteries of *α*-Sn*_x_*Fe_1−*x*_OOH NPs with ‘*x*’ of 0.0, 0.10, 0.15, and 0.20 (Sn100*x*) were investigated. From ^57^Fe- and ^119^Sn- Mössbauer spectra of Sn100*x* measured from 300 K to 20 K, we could successfully calculate the Debye temperature (*Θ*_D_). From the temperature-dependent ^57^Fe-Mössbauer spectra, the *Θ*_D_ of 210, 228, and 250 K were estimated for iron in Sn100*x* with ‘*x*’ of 0.10, 0.15, and 0.20, respectively. These *Θ*_D_ values are smaller than those of *α*-Fe_2_O_3_ (324 K), *γ*-Fe_2_O_3_ (300 K), and Fe_3_O_4_ (249 K). Similarly, the small *Θ*_D_ values of 199, 251, and 269 K compared with that of BaSnO_3_ (658 K) and SnO_2_ (382 K) were evaluated for tin in Sn100*x* from the ^119^Sn-Mössbauer spectra of the corresponding samples. These results evidently show that *Θ*_D_ values for iron and tin in Sn100*x* become smaller than the referenced iron and tin oxides due to the nanoformulations that occurred by the doping of tin. The impedance spectroscopy measurements indicate semiconducting nature with characteristic activation energy for studied tin-dopped *α*-Sn*_x_*Fe_1−*x*_OOH NPs. Modification of the NP results in an increase in DC conductivity for almost 5 orders of magnitude, σ_DC_ from 9.37 × 10^−7^ (Ω cm)^−1^ @423 K, up to a DC plateau of ~4 × 10^−7^ (Ω cm)^−1^ @423 K for samples with high Sn content, *x* = 0.15 and 0.20. The non-linear trend with a plateau suggests that structural modifications impact the charge transport which comes from the thermally activated via three-dimensional hopping of small polarons (SPH). The conductivity mechanism in studied NPs seems to be related to a combination of two dominant factors: (i) ease of electron/polaron mobility jumping along with (ii) the decrease in charge carrier concentration depending on the composition and Fe/Sn ratio. The observed plateau at highest Sn concentration indicates that the decrease in charge carriers overtakes the overall trend above *x* > 0.15 Sn NP. Due to the large electrical conductivity of Sn100*x*, the large initial capacities of 946 and 170 mAh g^−1^ were recorded from the Li- and Na-batteries containing Sn15 as the cathodes tested under the current density of 5.0 mA g^−1^. Further, the Sn15 cathode in the Na battery showed the largest discharge capacity of 85 mAh g^−1^ with a retention rate of 77% under the larger current density of 50 mA g^−1^. Due to the largest capacity and the retention rate, it is concluded that Sn15 is overall the best sample for a cathode active material for Na-battery compared with the other samples tested.

## Figures and Tables

**Table 1:** ^57^Fe-Mössbauer parameters of *α*-FeOOH NPs recorded from 300 to 20 K.

*T* (K)	*A* (%)	*δ* (mm s^−1^)	∆ (mm s^−1^)	*H*_int_ (T)	*Г* (mm s^−1^)	Phase
300	55.6	0.38_±0.03_	−0.19_±0.04_	31.0_±0.32_	1.26_±0.08_	Goethite
	38.2	0.37_±0.01_	−0.26_±0.02_	35.4_±0.07_	0.53_±0.04_	Goethite
	6.3	0.49_±0.01_	1.47_±0.02_	-	0.27_±0.03_	Superparamagnetic
250	49.5	0.38_±0.01_	−0.28_±0.01_	40.7_±0.04_	0.42_±0.02_	Goethite
	45.3	0.39_±0.02_	−0.25_±0.04_	37.3_±0.28_	0.99_±0.07_	Goethite
	5.2	0.54_±0.02_	1.75_±0.04_	-	0.32_±0.05_	Superparamagnetic
200	41.9	0.43_±0.01_	−0.26_±0.02_	41.7_±0.13_	0.70_±0.04_	Goethite
	58.1	0.41_±0.01_	−0.25_±0.01_	44.5_±0.03_	0.37_±0.01_	Goethite
150	51.8	0.44_±0.01_	−0.25_±0.01_	47.3_±0.08_	0.31_±0.01_	Goethite
	48.2	0.44_±0.01_	−0.24_±0.01_	45.5_±0.10_	0.53_±0.02_	Goethite
100	100	0.46_±0.01_	−0.25_±0.01_	48.7_±0.01_	0.43_±0.01_	Goethite
50	100	0.47_±0.01_	−0.24_±0.01_	49.7_±0.01_	0.46_±0.01_	Goethite
20	100	0.47_±0.01_	−0.24_±0.01_	50.0_±0.01_	0.48_±0.01_	Goethite

*A*: absorption area; *δ*: isomer shift; ∆: quadrupole splitting; *H*_int_: internal magnetic field; *Г*: full width at half maximum.

**Table 2:** ^57^Fe-Mössbauer parameters of *α*-Sn_0.10_Fe_0.90_OOH NPs recorded from 300 to 20 K.

*T* (K)	*A* (%)	*δ* (mm s^−1^)	∆ (mm s^−1^)	*H*_int_ (T)	*Г* (mm s^−1^)	Phase
300	100	0.33_±0.00_	−0.67_±0.01_	-	0.65_±0.01_	Superparamagnetic
250	100	0.37_±0.00_	0.69_±0.01_	-	0.58_±0.01_	Superparamagnetic
200	100	0.39_±0.00_	0.71_±0.01_	-	0.61_±0.01_	Superparamagnetic
150	100	0.42_±0.00_	0.72_±0.01_	-	0.73_±0.01_	Superparamagnetic
100	100	0.43_±0.01_	−0.76_±0.01_	-	0.93_±0.02_	Superparamagnetic
** 50	100	0.41_±0.02_	0.00_±158_	-	5.93_±0.41_	-
20	42.3	0.84_±0.04_	1.86_±0.10_	36.4_±0.23_	1.81_±0.12_	Unknown
	46.4	0.05_±0.04_	−1.78_±0.08_	37.2_±0.20_	1.76_±0.11_	Unknown
	11.3	0.57_±0.02_	2.04_±0.04_		0.80_±0.06_	Superparamagnetic

*A*: absorption area; *δ*: isomer shift; ∆: quadrupole splitting; *H*_int_: internal magnetic field; *Г*: full width at half maximum. ** Unable to analyze due to the low statistics.

**Table 3:** ^57^Fe-Mössbauer parameters of α-Sn_0.15_Fe_0.85_OOH NPs recorded from 300 to 20 K.

*T* (K)	*A* (%)	*δ* (mm s^−1^)	∆ (mm s^−1^)	*H*_int_ (T)	*Г* (mm s^−1^)	Phase
300	100	0.31_±0.01_	0.64_±0.01_	-	0.56_±0.01_	Superparamagnetic
250	26.5	0.23_±0.01_	−0.13_±0.30_	25.4_±1.20_	3.54_±0.79_	Unknown
	73.5	0.34_±0.01_	0.66_±0.01_	-	0.59_±0.01_	Superparamagnetic
200	38.1	0.39_±0.01_	−0.86_±0.37_	32.4_±1.52_	5.72_±1.15_	Goethite
	61.9	0.36_±0.01_	0.67_±0.01_	-	0.71_±0.01_	Superparamagnetic
150	27.0	0.51_±0.05_	−0.31_±0.08_	42.9_±0.30_	1.61_±0.13_	Goethite
	73.0	0.38_±0.01_	0.71_±0.01_	-	1.08_±0.02_	Superparamagnetic
100	15.1	0.48_±0.02_	−0.27_±0.04_	46.6_±0.12_	0.77_±0.07_	Goethite
	34.2	0.36_±0.01_	0.94_±0.04_	-	1.63_±0.17_	Superparamagnetic
	50.6	0.29_±0.08_	−5.35_±0.74_	-	6.3_±0.94_	Superparamagnetic
50	28.9	0.47_±0.01_	−0.20_±0.02_	48.1_±0.07_	0.70_±0.04_	Goethite
	63.1	0.47_±0.02_	−0.03_±0.04_	41.2_±0.26_	1.92_±0.08_	Goethite
	8.0	0.54_±0.03_	1.97_±0.06_	-	0.97_±0.09_	Superparamagnetic
20	52.1	0.46_±0.00_	−0.12_±0.01_	49.4_±0.04_	0.58_±0.02_	Goethite
	42.8	0.44_±0.01_	−0.06_±0.02_	45.8_±0.16_	0.93_±0.04_	Goethite
	5.2	0.54_±0.07_	1.86_±0.12_	-	1.15_±0.19_	Superparamagnetic

*A*: absorption area; *δ*: isomer shift; ∆: quadrupole splitting; *H*_int_: internal magnetic field; *Г*: full width at half maximum.

**Table 4:** ^57^Fe-Mössbauer parameters of *α*-Sn_0.20_Fe_0.80_OOH NPs recorded from 300 to 20 K.

*T* (K)	*A* (%)	*δ* (mm s^−1^)	∆ (mm s^−1^)	*H*_int_ (T)	*Г* (mm s^−1^)	Phase
300	100	0.32_±0.01_	0.66_±0.01_	-	0.57_±0.01_	Superparamagnetic
250	100	0.34_±0.01_	0.66_±0.01_	-	0.61_±0.01_	Superparamagnetic
200	100	0.36_±0.01_	0.68_±0.03_	-	0.69_±0.01_	Superparamagnetic
150	100	0.37_±0.01_	0.70_±0.01_	-	0.86_±0.01_	Superparamagnetic
100	22.0	0.52_±0.26_	−0.22_±0.31_	46.1_±0.96_	2.81_±0.70_	Goethite
	16.7	0.42_±0.11_	−0.20_±0.17_	24.5_±0.62_	1.74_±0.32_	Goethite
	61.3	0.32_±0.01_	0.92_±0.01_	-	1.13_±0.03_	Superparamagnetic
50	24.0	0.52_±0.06_	−0.22_±0.12_	46.1_±0.30_	1.62_±0.23_	Goethite
	45.1	0.42_±0.08_	−0.20_±0.16_	24.5_±1.22_	3.58_±0.95_	Goethite
	30.9	0.32_±0.02_	0.92_±0.10_	-	1.99_±0.30_	Superparamagnetic
20	41	0.53_±0.03_	−0.14_±0.06_	45.7_±0.17_	1.72_±0.12_	Goethite
	44.3	0.27_±0.07_	0.15_±0.11_	26.7_±0.42_	2.97_±0.42_	Unknown
	14.7	0.46_±0.03_	1.48_±0.05_	-	1.32_±0.10_	Superparamagnetic

*A*: absorption area; *δ*: isomer shift; ∆: quadrupole splitting; *H*_int_: internal magnetic field; *Г*: full width at half maximum.

**Table 5:** ^119^Sn-Mössbauer parameters of α-Sn_0.10_Fe_0.90_OOH NPs recorded from 300 to 20 K.

*T* (K)	A (%)	*δ* (mm s^−1^)	∆ (mm s^−1^)	*Г* (mm s^−1^)
300	100	0.08_±0.01_	0.49_±0.04_	0.96_±0.08_
250	100	0.09_±0.01_	0.50_±0.02_	0.89_±0.03_
200	100	0.09_±0.01_	0.47_±0.03_	1.06_±0.04_
150	100	0.08_±0.01_	0.23_±0. 14_	1.37_±0.06_
100	100	0.10_±0.01_	0.00_±8.85_	1.64_±0.07_
50	100	0.10_±0.01_	0.00_±27.3_	2.66_±0.13_
20	100	0.08_±0.02_	0.00_±6.23_	3.78_±0.23_

*A*: absorption area; *δ*: isomer shift; ∆: quadrupole splitting; *Г*: full width at half maximum.

**Table 6:** ^119^Sn-Mössbauer parameters of *α*-Sn_0.15_Fe_0.85_OOH NPs recorded from 300 to 20 K.

*T* (K)	*A* (%)	*δ* (mm s^−1^)	∆ (mm s^−1^)	*Г* (mm s^−1^)
300	100	0.09_±0.03_	0.64_±0.11_	1.42_±0.13_
250	100	0.10_±0.03_	0.63_±0.17_	1.75_±0.16_
200	100	0.09_±0.03_	0.00_±5.29_	2.51_±0.26_
150	100	0.12_±0.04_	0.00_±67.9_	2.89_±0.29_
100	100	0.17_±0.05_	0.00_±13.7_	3.98_±0.45_
50	100	0.13_±0.03_	1.28_±1.24_	2.78_±0.22_
20	100	0.24_±0.11_	3.52_±0.30_	6.06_±0.62_

*A*: absorption area; *δ*: isomer shift; ∆: quadrupole splitting; *Г*: full width at half maximum.

**Table 7:** ^119^Sn-Mössbauer parameters of *α*-Sn_0.20_Fe_0.80_OOH NPs recorded from 300 to 20 K.

*T* (K)	*A* (%)	*δ* (mm s^−1^)	∆ (mm s^−1^)	*Г* (mm s^−1^)
300	100	0.09_±0.02_	0.48_±0.13_	1.41_±0.11_
250	100	0.10_±0.02_	0.46_±0.15_	1.52_±0.12_
200	100	0.10_±0.02_	0.31_±0.27_	1.68_±0.13_
150	100	0.10_±0.02_	0.08_±1.28_	1.81_±0.13_
100	100	0.13_±0.02_	0.00_±27.2_	2.16_±0.15_
50	100	0.13_±0.03_	0.00_±50.0_	2.78_±0.22_
20	100	0.10_±0.03_	0.00_±76.7_	3.37_±0.29_

*A*: absorption area; *δ*: isomer shift; ∆: quadrupole splitting; *Г*: full width at half maximum.

**Table 8:** DC conductivity, *σ*_DC_; activation energy, *E*_DC_; and pre-exponential factor, $\sigma_{0}^{*}$ for *α*-Sn*_x_*Fe_1−*x*_OOH NPs series.

Sample	*σ*_DC_ @423 K (Ω^−1^ cm^−1^)	*E*_DC_ (kJ mol^−1^)	$\log\;\sigma_{0}^{*}$ (Ω^−1^ cm^−1^)
Sn0	1.45 × 10^−10^	93.9	1.56
Sn5	1.95 × 10^−8^	77.1	1.70
Sn10	1.12 × 10^−7^	74.9	2.20
Sn15	3.69 × 10^−7^	64.3	1.50
Sn20	4.27 × 10^−7^	45.5	−0.79

**Table 9:** Discharge capacity and capacity retention of the charge–discharge capacity performance for *α*-Sn*_x_*Fe_1−*x*_OOH NPs were measured at the current rates of 5 and 50 mAh g^−1^ in both LIBs and SIBs.

Sample Code	Initial Discharge Capacity (mAh g^−1^)				Capacity Retention (30 Cycles) (%)			
	5 mA g^−1^		50 mA g^−1^		5 mA g^−1^		50 mA g^−1^	
	SIB	LIB	SIB	LIB	SIB	LIB	SIB	LIB
Sn0	110	977	81	290	32	0.4	88	22
Sn5	77	-	43	-	60	-	90	-
Sn10	122	862	73	243	35	0.5	86	18
Sn15	170	946	85	347	27	0.1	77	9.6
Sn20	182	-	55	-	22	-	51	-

## Data Availability

The data presented in this study are available on request from the corresponding author.

## Supplementary Materials

The following supporting information can be downloaded at: https://www.mdpi.com/article/10.3390/ijms25052488/s1.
