# Supplementary material for: Debye Temperature Evaluation for Secondary Battery Cathode of α-SnxFe1−xOOH Nanoparticles Derived from the 57Fe- and 119Sn-Mössbauer Spectra
Source: Int J Mol Sci. 2024 Feb 20;25(5):2488. doi: 10.3390/ijms25052488 (PMC10931626; doi:10.3390/ijms25052488)
Supplement: Supplementary file 1 [file ijms-25-02488-s001.zip › ijms-2869847-supplementary.pdf]

# Supplementary Information

# Debye Temperature Evaluation for Secondary Battery Cathode of $\alpha$-$Sn_xFe_{1-x}OOH$ Nanoparticles Derived from the $^{57}Fe$- and $^{119}Sn$-Mössbauer Spectra

**Ahmed Ibrahim [1], Kaoru Tani [1], Kanae Hashi [1], Bofan Zhang [1], Zoltán Homonnay [2], Ernő Kuzmann [2], Arijeta Bafti [3], Luka Pavić [4], Stjepko Krehula [4], Marijan Marciuš [4] and Shiro Kubuki [1,*]**

[1] Department of Chemistry, Graduate School of Science, Tokyo Metropolitan University, Tokyo 192-0397, Japan

[2] Institute of Chemistry, Eötvos Loránd University, 1117 Budapest, Hungary

[3] Faculty of Chemical Engineering and Technology, University of Zagreb, 10000 Zagreb, Croatia

[4] Division of Materials Chemistry, Ruđer Bošković Institute, 10000 Zagreb, Croatia

*Correspondence: kubuki@tmu.ac.jp

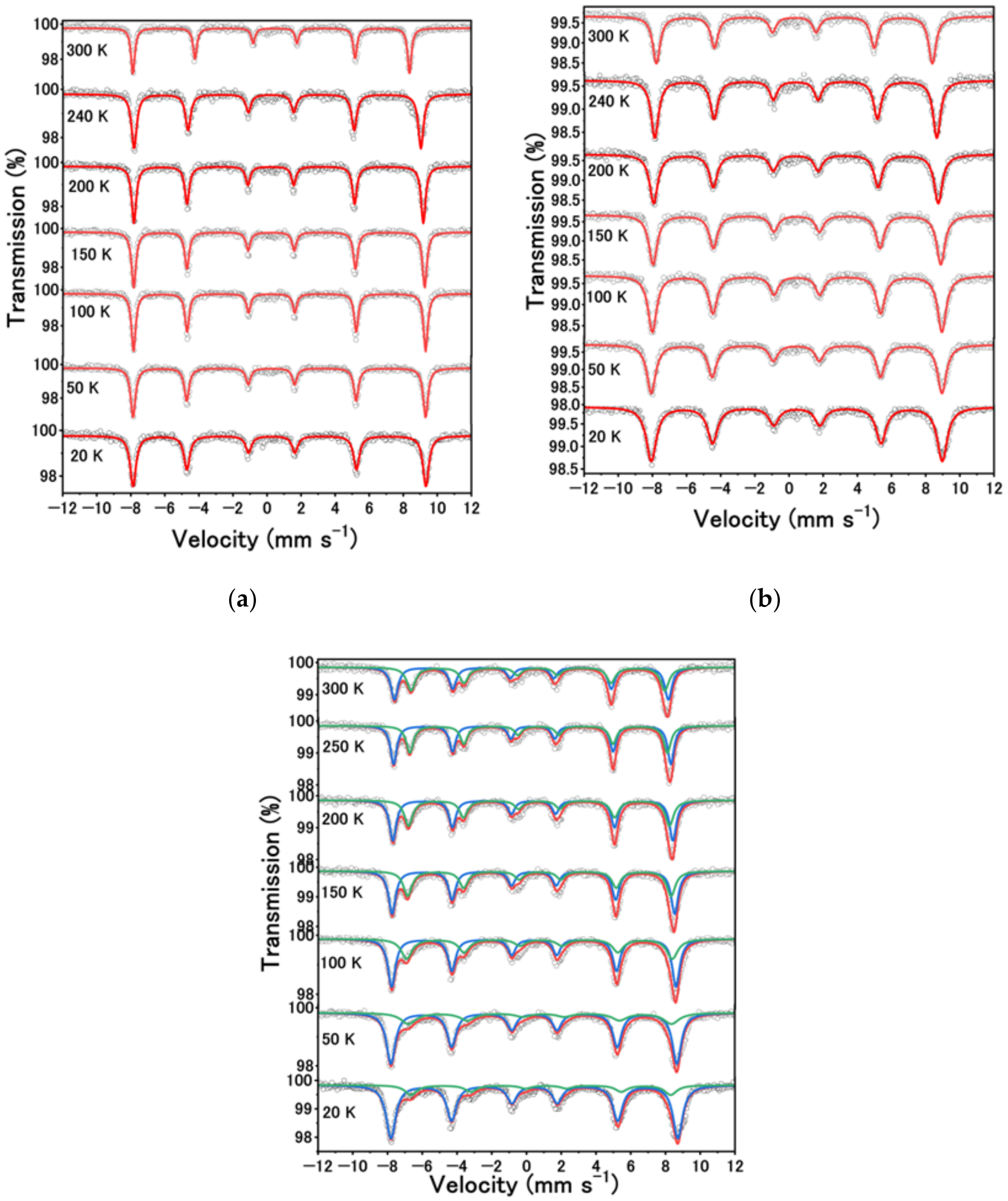


(a) (b) (c)

**Figure S1.** [57]Fe-Mössbauer spectra of $\alpha$-$Fe_2O_3$ (**a**), $\gamma$-$Fe_2O_3$ (**b**), and $Fe_3O_4$ (**c**) measured at 300, 250, 200, 150, 100, 50, 20 K.

**Table S1.** $^{57}$Fe-Mössbauer parameters of $\alpha$-$Fe_2O_3$ recorded from 300 to 20 K.

| $T$ (K) | $\delta$ (mm s$^{-1}$) | $\Delta$ (mm s$^{-1}$) | $H_{int}$ (T) | $\Gamma$ (mm s$^{-1}$) |
|---|---|---|---|---|
| 300 | $0.368_{\pm 0.01}$ | $-0.227_{\pm 0.01}$ | $50.50_{\pm 0.01}$ | $0.269_{\pm 0.01}$ |
| 260 | $0.399_{\pm 0.01}$ | $-0.179_{\pm 0.01}$ | $51.11_{\pm 0.02}$ | $0.312_{\pm 0.01}$ |
| 240 | $0.414_{\pm 0.01}$ | $0.376_{\pm 0.01}$ | $52.37_{\pm 0.01}$ | $0.372_{\pm 0.01}$ |
| 220 | $0.425_{\pm 0.01}$ | $0.441_{\pm 0.01}$ | $52.62_{\pm 0.01}$ | $0.311_{\pm 0.01}$ |
| 200 | $0.440_{\pm 0.01}$ | $0.459_{\pm 0.01}$ | $52.84_{\pm 0.01}$ | $0.321_{\pm 0.01}$ |
| 150 | $0.468_{\pm 0.01}$ | $0.469_{\pm 0.01}$ | $53.13_{\pm 0.01}$ | $0.313_{\pm 0.01}$ |
| 100 | $0.492_{\pm 0.01}$ | $0.467_{\pm 0.01}$ | $53.33_{\pm 0.01}$ | $0.297_{\pm 0.01}$ |
| 50 | $0.499_{\pm 0.01}$ | $0.465_{\pm 0.01}$ | $53.42_{\pm 0.01}$ | $0.339_{\pm 0.01}$ |
| 20 | $0.503_{\pm 0.01}$ | $0.458_{\pm 0.01}$ | $53.45_{\pm 0.02}$ | $0.415_{\pm 0.01}$ |

$\delta$: isomer shift, $\Delta$: quadrupole splitting, $H_{int}$: internal magnetic field, $\Gamma$: full width at half maximum.

**Table S2.** $^{57}$Fe-Mössbauer parameters of $\gamma$-$Fe_2O_3$ recorded from 300 to 20 K.

| $T$ (K) | $\delta$ (mm s$^{-1}$) | $\Delta$ (mm s$^{-1}$) | $H_{int}$ (T) | $\Gamma$ (mm s$^{-1}$) |
|---|---|---|---|---|
| 300 | $0.319_{\pm 0.01}$ | $0.003_{\pm 0.007}$ | $50.24_{\pm 0.03}$ | $0.52_{\pm 0.01}$ |
| 280 | $0.333_{\pm 0.01}$ | $0.015_{\pm 0.007}$ | $50.67_{\pm 0.02}$ | $0.53_{\pm 0.01}$ |
| 260 | $0.344_{\pm 0.01}$ | $0.012_{\pm 0.007}$ | $51.04_{\pm 0.03}$ | $0.56_{\pm 0.01}$ |
| 240 | $0.383_{\pm 0.01}$ | $0.004_{\pm 0.007}$ | $51.34_{\pm 0.03}$ | $0.53_{\pm 0.01}$ |
| 220 | $0.377_{\pm 0.01}$ | $0.004_{\pm 0.008}$ | $51.62_{\pm 0.03}$ | $0.57_{\pm 0.01}$ |
| 200 | $0.386_{\pm 0.01}$ | $0.016_{\pm 0.007}$ | $51.82_{\pm 0.02}$ | $0.58_{\pm 0.01}$ |
| 150 | $0.441_{\pm 0.01}$ | $0.014_{\pm 0.007}$ | $52.39_{\pm 0.02}$ | $0.60_{\pm 0.01}$ |
| 100 | $0.451_{\pm 0.01}$ | $0.019_{\pm 0.007}$ | $52.71_{\pm 0.02}$ | $0.61_{\pm 0.01}$ |
| 50 | $0.444_{\pm 0.01}$ | $0.016_{\pm 0.006}$ | $52.92_{\pm 0.02}$ | $0.63_{\pm 0.01}$ |
| 20 | $0.453_{\pm 0.01}$ | $-0.000_{\pm 0.009}$ | $52.95_{\pm 0.03}$ | $0.71_{\pm 0.01}$ |

$\delta$: isomer shift, $\Delta$: quadrupole splitting, $H_{int}$: internal magnetic field, $\Gamma$: full width at half maximum.

**Table S3.** $^{57}$Fe-Mössbauer parameters of $Fe_3O_4$ recorded from 300 to 20 K.

| ***T*** **(K)** | ***A*** **(%)** | ***δ*** **(mm s⁻¹)** | ***Δ*** **(mm s⁻¹)** | ***H*int (T)** | ***Γ*** **(mm s⁻¹)** |
|---|---|---|---|---|---|
| 300 | 53.8 | $0.311_{\pm 0.01}$ | $-0.038_{\pm 0.01}$ | $49.02_{\pm 0.04}$ | $0.73_{\pm 0.02}$ |
| | 46.2 | $0.655_{\pm 0.01}$ | $0.02_{\pm 0.02}$ | $45.43_{\pm 0.06}$ | $0.71_{\pm 0.03}$ |
| 250 | 55.2 | $0.334_{\pm 0.01}$ | $-0.032_{\pm 0.01}$ | $49.62_{\pm 0.03}$ | $0.44_{\pm 0.01}$ |
| | 44.8 | $0.678_{\pm 0.01}$ | $0.01_{\pm 0.01}$ | $46.09_{\pm 0.04}$ | $0.50_{\pm 0.02}$ |
| 200 | 56.4 | $0.372_{\pm 0.01}$ | $-0.034_{\pm 0.01}$ | $50.12_{\pm 0.03}$ | $0.47_{\pm 0.01}$ |
| | 41.3 | $0.744_{\pm 0.01}$ | $-0.03_{\pm 0.01}$ | $47.16_{\pm 0.05}$ | $0.60_{\pm 0.02}$ |
| 150 | 58.7 | $0.399_{\pm 0.01}$ | $-0.028_{\pm 0.01}$ | $50.56_{\pm 0.02}$ | $0.49_{\pm 0.01}$ |
| | 41.3 | $0.744_{\pm 0.01}$ | $-0.03_{\pm 0.01}$ | $47.16_{\pm 0.05}$ | $0.60_{\pm 0.02}$ |
| 100 | 62.1 | $0.422_{\pm 0.01}$ | $-0.023_{\pm 0.01}$ | $50.88_{\pm 0.02}$ | $0.52_{\pm 0.01}$ |
| | 37.9 | $0.78_{\pm 0.01}$ | $-0.09_{\pm 0.02}$ | $47.55_{\pm 0.07}$ | $0.76_{\pm 0.03}$ |
| 80 | 66.1 | $0.442_{\pm 0.01}$ | $-0.032_{\pm 0.01}$ | $51.05_{\pm 0.02}$ | $0.56_{\pm 0.01}$ |
| | 33.9 | $0.80_{\pm 0.01}$ | $-0.07_{\pm 0.02}$ | $47.35_{\pm 0.09}$ | $0.83_{\pm 0.04}$ |
| 50 | 72.9 | $0.448_{\pm 0.01}$ | $-0.033_{\pm 0.01}$ | $51.17_{\pm 0.02}$ | $0.62_{\pm 0.01}$ |
| | 27.1 | $0.88_{\pm 0.02}$ | $-0.20_{\pm 0.04}$ | $47.10_{\pm 0.20}$ | $1.07_{\pm 0.07}$ |
| 20 | 81 | $0.462_{\pm 0.01}$ | $-0.015_{\pm 0.01}$ | $51.33_{\pm 0.02}$ | $0.66_{\pm 0.01}$ |
| | 19 | $0.97_{\pm 0.02}$ | $-0.30_{\pm 0.04}$ | $46.50_{\pm 0.10}$ | $0.84_{\pm 0.06}$ |

*A*: absorption area *δ*: isomer shift, *Δ*: quadrupole splitting, $H_{int}$: internal magnetic field, *Γ*: full width at half maximum.

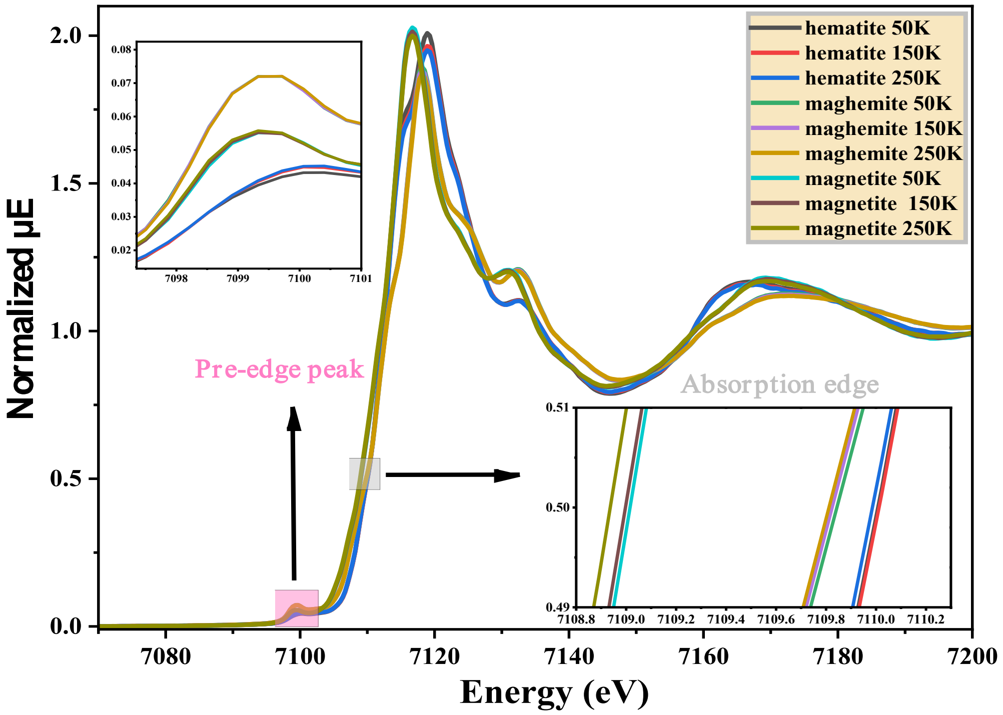


(a)

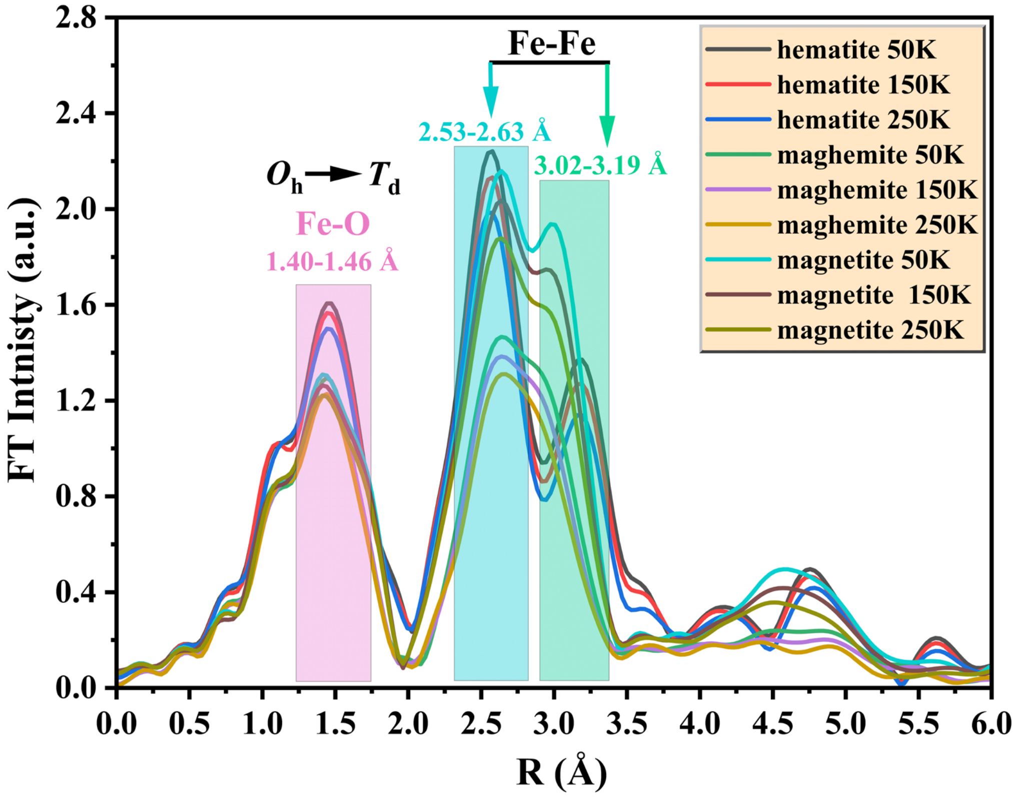


(b)

**Figure S2.** (**a**) XANES and (**b**) EXAFS spectra of hematite, maghemite, and magnetite measured at 50, 150, and 250 K.

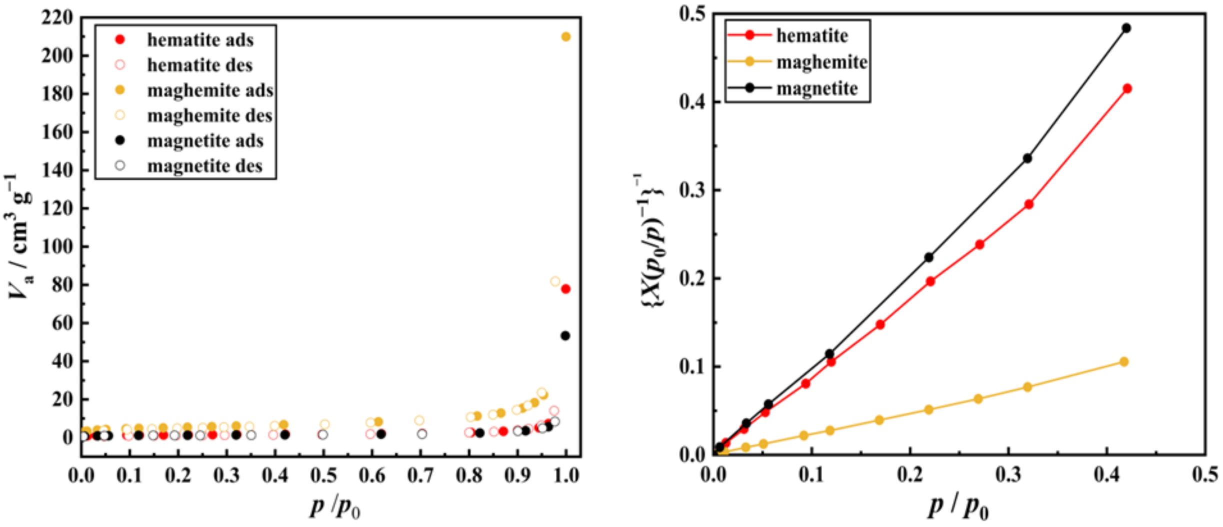


(**a**) (**b**)

**Figure S3.** BET and $V$a($p_0$-$p$) vs. $p$/$p_0$ plot of hematite, maghemite and magnetite.

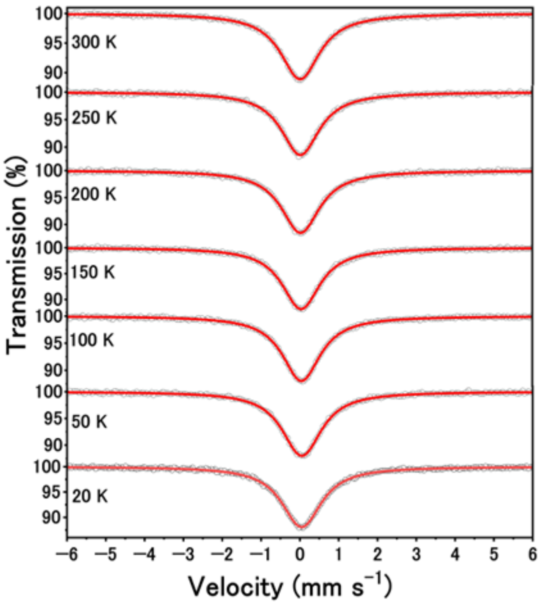


(**a**)

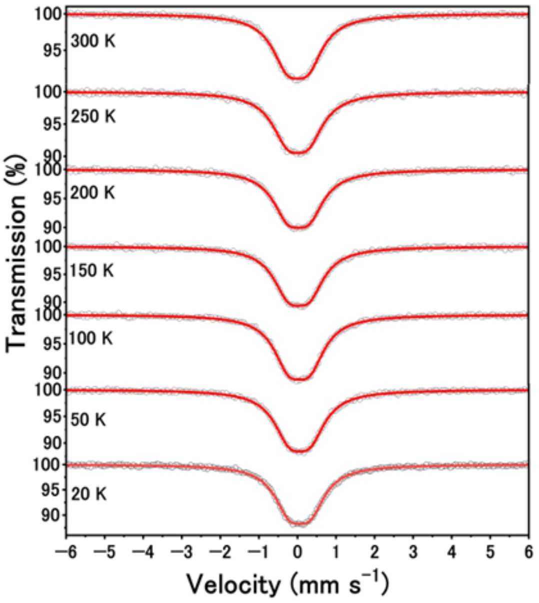


(**b**)

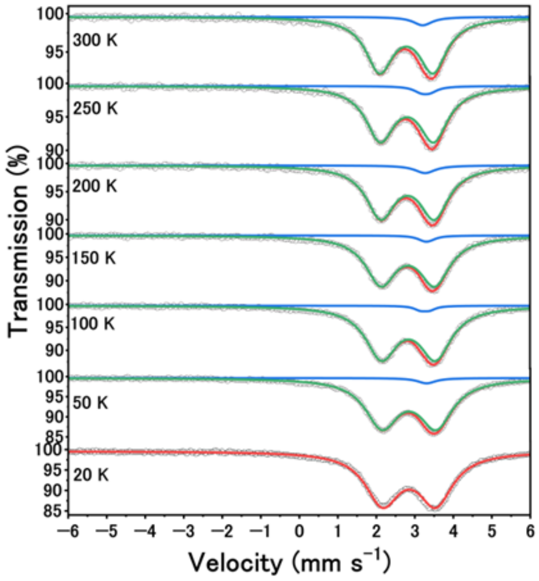


(**c**)

**Figure S4.** $^{119}$Sn-Mössbauer spectra of (**a**) $BaSnO_3$, (**b**) $SnO_2$ and (**c**) SnO measured at 300, 250, 200, 150, 100, 50, 20 K.

**Table S4.** $^{119}$Sn-Mössbauer parameters of $BaSnO_3$ recorded from 300 to 20 K.

| *T* (K) | *A* (%) | *δ* (mm s$^{-1}$) | *Δ* (mm s$^{-1}$) | *Γ* (mm s$^{-1}$) |
|---|---|---|---|---|
| 300 | 100 | $0.00_{\pm 0.00}$ | $0.01_{\pm 18.65}$ | $1.23_{\pm 0.02}$ |
| 280 | 100 | $0.02_{\pm 0.00}$ | $0.00_{\pm 17.37}$ | $1.20_{\pm 0.02}$ |
| 260 | 100 | $0.02_{\pm 0.00}$ | $0.00_{\pm 1.72}$ | $1.21_{\pm 0.02}$ |
| 240 | 100 | $0.04_{\pm 0.00}$ | $0.11_{\pm 0.07}$ | $1.18_{\pm 0.02}$ |
| 220 | 100 | $0.04_{\pm 0.00}$ | $0.00_{\pm 1.64}$ | $1.20_{\pm 0.02}$ |
| 200 | 100 | $0.05_{\pm 0.00}$ | $0.20_{\pm 0.04}$ | $1.18_{\pm 0.02}$ |
| 150 | 100 | $0.05_{\pm 0.00}$ | $0.16_{\pm 0.05}$ | $1.21_{\pm 0.02}$ |
| 100 | 100 | $0.00_{\pm 0.00}$ | $0.01_{\pm 18.65}$ | $1.23_{\pm 0.02}$ |
| 50 | 100 | $0.02_{\pm 0.00}$ | $0.00_{\pm 17.37}$ | $1.20_{\pm 0.02}$ |
| 20 | 100 | $0.02_{\pm 0.00}$ | $0.00_{\pm 1.72}$ | $1.21_{\pm 0.02}$ |

*A*: absorption area,*δ*: isomer shift, *Δ*: quadrupole splitting, *Γ*: full width at half maximum.

**Table S5.** $^{119}$Sn-Mössbauer parameters of $SnO_2$ recorded from 300 to 20 K.

| *T* (K) | *A* (%) | *δ* (mm s$^{-1}$) | *Δ* (mm s$^{-1}$) | *Γ* (mm s$^{-1}$) |
|---|---|---|---|---|
| 300 | 100 | $0.02_{\pm 0.00}$ | $0.54_{\pm 0.01}$ | $0.96_{\pm 0.01}$ |
| 250 | 100 | $0.03_{\pm 0.00}$ | $0.54_{\pm 0.01}$ | $0.98_{\pm 0.01}$ |
| 200 | 100 | $0.05_{\pm 0.00}$ | $0.55_{\pm 0.01}$ | $0.96_{\pm 0.01}$ |
| 150 | 100 | $0.06_{\pm 0.00}$ | $0.54_{\pm 0.01}$ | $0.95_{\pm 0.01}$ |
| 100 | 100 | $0.07_{\pm 0.00}$ | $0.54_{\pm 0.01}$ | $0.95_{\pm 0.01}$ |
| 50 | 100 | $0.07_{\pm 0.00}$ | $0.53_{\pm 0.01}$ | $0.97_{\pm 0.01}$ |
| 20 | 100 | $0.08_{\pm 0.00}$ | $0.53_{\pm 0.01}$ | $0.94_{\pm 0.01}$ |

*A*: absorption area,*δ*: isomer shift, *Δ*: quadrupole splitting, *Γ*: full width at half maximum.

**Table S6.** $^{119}$Sn-Mössbauer parameters of SnO recorded from 300 to 20 K.

| *T* (K) | *A* (%) | *δ* (mm s$^{-1}$) | *Δ* (mm s$^{-1}$) | *Γ* (mm s$^{-1}$) |
|---|---|---|---|---|
| 300 | 94.8 | $2.79_{\pm 0.01}$ | $1.39_{\pm 0.01}$ | $0.88_{\pm 0.01}$ |
| | 5.2 | $3.22_{\pm 0.03}$ | $0.00_{\pm 2}$ | $0.60_{\pm 0.40}$ |
| 250 | 95.1 | $2.80_{\pm 0.01}$ | $1.39_{\pm 0.01}$ | $0.91_{\pm 0.01}$ |
| | 4.9 | $3.28_{\pm 0.03}$ | $0.22_{\pm 0.07}$ | $0.50_{\pm 0.10}$ |
| 200 | 94.9 | $2.82_{\pm 0.01}$ | $1.39_{\pm 0.01}$ | $0.92_{\pm 0.01}$ |
| | 5.1 | $3.27_{\pm 0.03}$ | $0.00_{\pm 10}$ | $0.60_{\pm 0.30}$ |
| 150 | 96.8 | $2.84_{\pm 0.01}$ | $1.39_{\pm 0.01}$ | $0.97_{\pm 0.01}$ |
| | 3.2 | $3.31_{\pm 0.02}$ | $0.10_{\pm 0.10}$ | $0.40_{\pm 0.10}$ |
| 100 | 97.2 | $2.84_{\pm 0.01}$ | $1.40_{\pm 0.01}$ | $1.01_{\pm 0.01}$ |
| | 2.8 | $3.27_{\pm 0.02}$ | $0.24_{\pm 0.03}$ | $0.38_{\pm 0.07}$ |
| 50 | 97.0 | $2.85_{\pm 0.01}$ | $1.41_{\pm 0.01}$ | $1.06_{\pm 0.01}$ |
| | 3.0 | $3.31_{\pm 0.02}$ | $0.00_{\pm 10}$ | $0.60_{\pm 0.30}$ |
| 20 | 100 | $2.85_{\pm 0.01}$ | $1.38_{\pm 0.01}$ | $1.09_{\pm 0.01}$ |

*A*: absorption area,*δ*: isomer shift, *Δ*: quadrupole splitting, *Γ*: full width at half maximum.

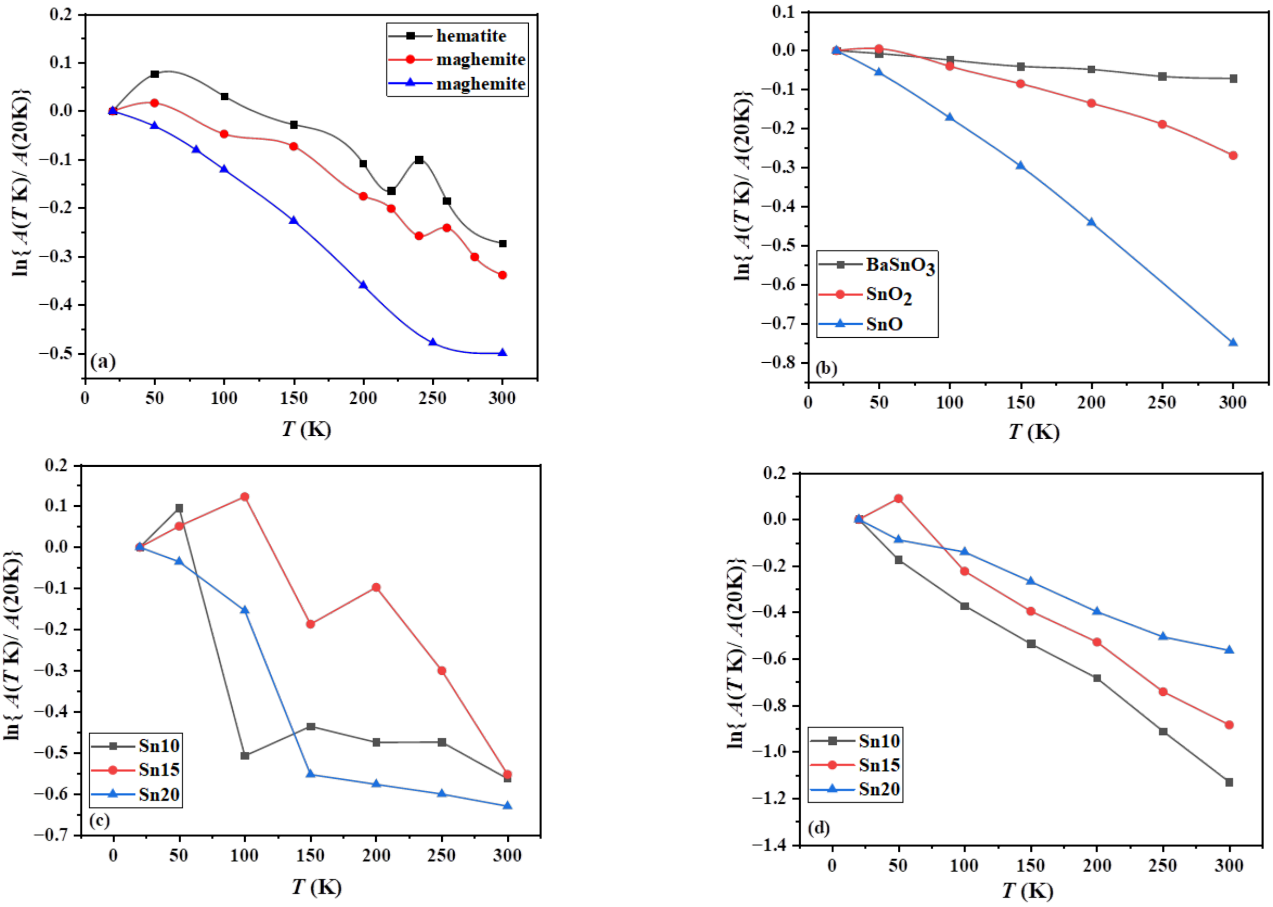


**Figure S5.** The temperature dependence of the normalized absorption area for iron oxide, tin compounds and $\alpha$-Sn$_x$Fe$_{1-x}$OOH NPs derived from the and 57Fe- and 119Sn-Mössbauer spectra of $\alpha$-Sn$_x$Fe$_{1-x}$OOH NPs with '$x$' of 0.10, 0.15 and 0.20 measured at 300, 250, 200, 150, 100, 50, 20 K.
